# CoMM-S^4^: A Collaborative Mixed Model Using Summary-Level eQTL and GWAS Datasets in Transcriptome-Wide Association Studies

**DOI:** 10.3389/fgene.2021.704538

**Published:** 2021-09-20

**Authors:** Yi Yang, Kar-Fu Yeung, Jin Liu

**Affiliations:** Centre for Quantitative Medicine, Program in Health Services and Systems Research, Duke-NUS Medical School, Singapore, Singapore

**Keywords:** summary statistics, genome-wide association studies, variational bayesian, parameter expanded expectation-maximization (PX-EM) algorithm, transcriptome-wide association studies

## Abstract

**Motivation:** Genome-wide association studies (GWAS) have achieved remarkable success in identifying SNP-trait associations in the last decade. However, it is challenging to identify the mechanisms that connect the genetic variants with complex traits as the majority of GWAS associations are in non-coding regions. Methods that integrate genomic and transcriptomic data allow us to investigate how genetic variants may affect a trait through their effect on gene expression. These include CoMM and CoMM-S^2^, likelihood-ratio-based methods that integrate GWAS and eQTL studies to assess expression-trait association. However, their reliance on individual-level eQTL data render them inapplicable when only summary-level eQTL results, such as those from large-scale eQTL analyses, are available.

**Result:** We develop an efficient probabilistic model, CoMM-S^4^, to explore the expression-trait association using summary-level eQTL and GWAS datasets. Compared with CoMM-S^2^, which uses individual-level eQTL data, CoMM-S^4^ requires only summary-level eQTL data. To test expression-trait association, an efficient variational Bayesian EM algorithm and a likelihood ratio test were constructed. We applied CoMM-S^4^ to both simulated and real data. The simulation results demonstrate that CoMM-S^4^ can perform as well as CoMM-S^2^ and S-PrediXcan, and analyses using GWAS summary statistics from Biobank Japan and eQTL summary statistics from eQTLGen and GTEx suggest novel susceptibility loci for cardiovascular diseases and osteoporosis.

**Availability and implementation:** The developed R package is available at https://github.com/gordonliu810822/CoMM.

## 1 Introduction

Genome-wide association studies (GWAS) have identified a large number of genetic risk variants associated with complex traits, with over 250,000 single nucleotide polymorphism (SNP)-trait associations tagged as significant in the NHGRI-EBI GWAS Catalog (Buniello et al., 2018). However, the specific biological mechanisms through which the identified genetic variants affect these traits have yet to be elucidated. Genetic variants may influence complex traits by altering gene expression and, consequently, protein abundance. These genetic variants may be within the regulatory sequences or secondary motifs of the target gene (cis regulation), or may affect genes at larger genomic distances by modifying upstream regulators which interact with the *cis*-regulatory sequences (Williams et al., 2007).

Transcriptome-wide association studies (TWAS) aim to provide insights into the specific mechanisms through which variants affect traits. In TWAS, the gene expression of GWAS samples is predicted with the aid of an eQTL dataset; the predicted expression is then analysed for any association with the trait of interest. Unlike approaches that examine gene expression and genetic variants in a pairwise manner, TWAS consider the combinatory effects of all genetic variants within a pre-defined window of the target gene, hence it is especially effective at detecting novel susceptibility loci when multiple variants influence expression. TWAS have proved useful as a stepping stone to generate new insights to a range of complex traits, including schizophrenia (Gusev et al., 2018), glioma (Strunz et al., 2020), prostate cancer (Mancuso et al., 2018), and age-related macular degeneration (Atkins et al., 2019).

Existing TWAS methods can be categorised into two groups, depending on whether they use individual-level or summary-level GWAS data. PrediXcan (Gamazon et al., 2015) and CoMM (Yang et al., 2018) use individual-level GWAS data, while S-PrediXcan (Barbeira et al., 2018) and CoMM-S^2^ (Yang et al., 2020) use summary-level GWAS data in conjunction with a matching reference panel to estimate linkage disequilibrium. Both CoMM and CoMM-S^2^ account for the imputation uncertainty in the prediction step and thus are more powerful in identifying expression-trait associations than other methods. However, these methods are limited by the availability of individual-level transcriptome data, and they neglect the ready accessibility of summary-level eQTL datasets. Datasets of eQTL summary statistics are maintained by various consortia including the eQTLGen Consortium (Võsa et al., 2018) and the GTEx Consortium (The GTEx Consortium, 2020). The ability to integrate summary-level eQTL data and summary-level GWAS data would broaden the scope of studies to which TWAS can be applied.

Here we introduce a powerful strategy that integrates eQTL summary statistics (SNP-expression correlation), GWAS summary statistics (SNP-phenotype correlation), and linkage disequilibrium information from reference panels (SNP-SNP correlation) to assess the association between the *cis* component of expression and trait. We extend CoMM-S^2^, a likelihood-based method which uses individual-level eQTL data to assess expression-trait association, and propose a probabilistic model, Collaborative Mixed Models using Summary Statistics from eQTL and GWAS (CoMM-S^4^). Compared with CoMM-S^2^, a major advantage of CoMM-S^4^ is its ability to use summary-level eQTL data and integrate them with GWAS summary statistics. In CoMM-S^4^, a joint likelihood is constructed using summary statistics from GWAS and eQTL studies, as well as SNP correlation information from reference panels representative of the GWAS and eQTL populations. We develop an efficient algorithm based on variational Bayes expectation-maximization and parameter expansion (PX-VBEM). To examine the expression-trait association, a likelihood ratio test is constructed.

The performance of CoMM-S^4^ is assessed in simulated data, and is also applied to traits from the NFBC1966 cohort (Sabatti et al., 2009) and Biobank Japan (Ishigaki et al., 2020). The TWAS analysis using GWAS summary statistics from NFBC1966 and eQTL summary statistics from eQTLGen suggest novel susceptibility loci for lipid traits, glucose levels, insulin levels and C-reactive protein, when compared against known susceptibility loci in the GWAS Catalog (Buniello et al., 2018). Moreover, the TWAS analysis using GWAS summary statistics from Biobank Japan and eQTL summary statistics from eQTLGen and GTEx reiterate the importance of MHC molecules, interferon-gamma signalling and apoptosis for several autoimmune and infection-related traits (rheumatoid arthritis, Graves’ disease, chronic hepatitis B and chronic hepatitis C), and suggest novel susceptibility loci for cardiovascular traits (congestive heart failure, ischemic stroke, peripheral artery disease) and osteoporosis.

## 2 Materials and Methods

### 2.1 Notation

We denote the individual-level eQTL dataset for *n*_1_ samples by {**Y**, **W**_1_}, where **Y** is the gene expression matrix for *g* genes and **W**_1_ is the genotype matrix for *m* SNP positions. For the *j*-th gene, let **y**_*j*_ denote the gene expression vector, and $W_{1j}\in R^{n_{1}\times m_{j}}$ denote the centered genotype matrix for the *m*_*j*_ SNPs within a pre-defined distance from the gene. In addition, we denote the individual-level GWAS dataset for *n*_2_ samples by {**z**, **W**_2_}, where **z** is the phenotype vector and **W**_2_ is the genotype matrix. Similarly, for the *j*-th gene, $W_{2j}\in R^{n_{2}\times m_{j}}$ denotes the centered genotype matrix for the *m*_*j*_ SNPs within a pre-defined distance from the gene.

We have the summary statistics, in the form of z-scores, from the analysis of genetic variant-gene expression pairs in the eQTL dataset. We also have the summary statistics from single-variate analysis in the GWAS dataset. We denote the eQTL z-scores for the *j*-th gene by ${\hat{\gamma }}_{1j}\in R^{m_{j}}$, and the GWAS z-scores by ${\hat{\gamma }}_{2j}\in R^{m_{j}}$ (*j* = 1, *…* , *g*). To model linkage disequilibrium (LD) in the eQTL and GWAS datasets, we require the SNP correlation matrices ${\hat{R}}_{1j}\in R^{m_{j}\times m_{j}}$ and ${\hat{R}}_{2j}\in R^{m_{j}\times m_{j}}$ (*j* = 1, *…* , *g*) estimated using reference panels that correspond to the eQTL and GWAS populations respectively.

### 2.2 Model

The relationship between the *j*-th gene expression **y**_*j*_ and genotype **W**_1*j*_ is modelled as

$$y_{j}=W_{1j}\beta_{1j}+e_{1},$$ (1)

where $\beta_{1j}={\left[\beta_{1j,1},\ldots ,\beta_{1j,m_{j}}\right]}^{T}$ is an *m*_*j*_-vector of effect sizes, and $e_{1}∼N\left(0,\sigma_{e_{1}}^{2}I\right)$ is an *n*_1_-vector of independent noise. Similarly, the relationship between trait **z** and genotype **W**_2*j*_ is modelled as

$$z=W_{2j}\beta_{2j}+e_{2},$$ (2)

where $\beta_{2j}={\left[\beta_{2j,1},\ldots ,\beta_{2j,m_{j}}\right]}^{T}$ is an *m*_*j*_-vector of effect sizes, and $e_{2}∼N\left(0,\sigma_{e_{2}}^{2}I\right)$ is an *n*_2_-vector of independent noise. We further model the GWAS effect size as ***β***_2*j*_ = *α*_*j*_ ***β***_1*j*_, where *α*_*j*_ can be interpreted as the effect of gene expression on phenotype under the assumption of no horizontal pleiotropy. To perform a likelihood ratio test for the null hypothesis *α*_*j*_ = 0, we first derive the form of the log-likelihood and develop an efficient algorithm to estimate its parameters.

Let ${\hat{\gamma }}_{1j}={\left[{\hat{\gamma }}_{1j,1},\ldots ,{\hat{\gamma }}_{1j,m_{j}}\right]}^{T}$ and ${\hat{\gamma }}_{2j}={\left[{\hat{\gamma }}_{2j,1},\ldots ,{\hat{\gamma }}_{2j,m_{j}}\right]}^{T}$ denote the z-scores for the eQTL and GWAS data, respectively. Let ${\hat{s}}_{1j}={\left[{\hat{s}}_{1j,1},\ldots ,{\hat{s}}_{1j,m_{j}}\right]}^{T}$ and ${\hat{s}}_{2j}={\left[{\hat{s}}_{2j,1},\ldots ,{\hat{s}}_{2j,m_{j}}\right]}^{T}$ denote the standard errors of the effect size estimators, ${\hat{\beta }}_{1j}$ and ${\hat{\beta }}_{2j}$, in the eQTL and GWAS analyses respectively. Using the approximated likelihood in regression with summary statistics (RSS) (Zhu and Stephens, 2017), the distribution for ${\hat{\beta }}_{ij}$ can be written as ${\hat{\beta }}_{ij}|\beta_{ij},{\hat{R}}_{ij}$, ${\hat{S}}_{ij}∼N\left({\hat{S}}_{ij}{\hat{R}}_{ij}{\hat{S}}_{ij}^{-1}\beta_{ij},{\hat{S}}_{ij}{\hat{R}}_{ij}{\hat{S}}_{ij}\right)$, where ${\hat{S}}_{ij}=\text{diag}\left({\hat{s}}_{ij}\right)$ (*i* = 1, 2). Details regarding this approximated distribution can also be found in related literature (Hormozdiari et al., 2014; Huang et al., 2021). In practice, we may observe only the z-scores for the summary statistics. In this case, the distribution of the eQTL z-scores ${\hat{\gamma }}_{1j}={\hat{S}}_{1j}^{-1}{\hat{\beta }}_{1j}$ can be written as

$${\hat{\gamma }}_{1j}|\gamma_{j},{\hat{R}}_{1j}∼N\left({\hat{R}}_{1j}\gamma_{j},{\hat{R}}_{1j}\right),$$ (3)

where $\gamma_{j}={\hat{S}}_{1j}^{-1}\beta_{1j}$. Similarly, the distribution of the GWAS z-scores ${\hat{\gamma }}_{2j}$ can be approximated by

$${\hat{\gamma }}_{2j}|\gamma_{j},{\hat{R}}_{2j}∼N\left(\alpha_{j}c_{j}{\hat{R}}_{2j}\gamma_{j},{\hat{R}}_{2j}\right),$$ (4)

where $c_{j}\approx \frac{{\hat{\sigma }}_{yj}}{{\hat{\sigma }}_{z}}\sqrt{\frac{n_{2}}{n_{1}}}$ when the summary statistics are generated using simple linear regression, ${\hat{\sigma }}_{yj}$ is the sample standard deviation for the expression of gene *j*, and ${\hat{\sigma }}_{z}$ is the sample standard deviation of the trait (details in Supplementary Material). Furthermore, a Gaussian prior is used for ***γ***_*j*_,

$$\gamma_{j}∼N\left(0,\sigma_{\gamma_{j}}^{2}I_{m_{j}}\right),$$ (5)

and the complete-data likelihood can be written as

$$\text{Pr}\left({\hat{\gamma }}_{1j},{\hat{\gamma }}_{2j},\gamma_{j}|{\hat{R}}_{1j},{\hat{R}}_{2j};\theta \right)=\overset{2}{\underset{i=1}{\prod }}\text{pr}\left({\hat{\gamma }}_{ij}|\gamma_{j},{\hat{R}}_{ij}\right)\text{pr}\left(\gamma_{j}\right),$$ (6)

where $\theta =\left\{\sigma_{\gamma_{j}}^{2},\alpha_{j}^{\prime }\right\}$ is the collection of parameters and $\alpha_{j}^{\prime }=\alpha_{j}c_{j}$.

We are primarily interested in the effect *α*_*j*_ of gene expression on trait. Notably, testing the hypothesis of whether *α*_*j*_ = 0 is equivalent to testing whether $\alpha_{j}^{\prime }=0$, as *c*_*j*_ is a positive constant. The accuracy of the above distributional approximations depend on the sample sizes of the eQTL and GWAS datasets, as well as the number of SNPs/genes associated with the gene expression/phenotype. The larger the sample size and the higher the degree of polygenecity, the greater the estimation accuracy.

### 2.3 Parameter Expansion-Variational Bayes Expectation-Maximization Algorithm

An efficient algorithm is needed to estimate the parameters of the model. Although the EM algorithm is widely used and has a highly stable performance, it requires inverting the matrix ${\hat{R}}_{1j}$ and ${\hat{R}}_{2j}$ in each iteration. To speed up the computational process, we use Variational Bayes Expectation-Maximization (VBEM), augmented with parameter expansion (PX) (Liu et al., 1998). The parameter-expanded model is

$${\hat{\gamma }}_{1j}|\gamma_{j},{\hat{R}}_{1j}∼N\left(\tau {\hat{R}}_{1j}\gamma_{j},{\hat{R}}_{1j}\right),$$ (7)

where the τ∈R is the expanded parameter. The model parameters are $\theta =\left\{\sigma_{\gamma_{j}}^{2},\alpha_{j},\tau \right\}$, and the expanded model reduces to the original one when *τ* = 1. In VBEM, the marginal log-likelihood can be decomposed into the evidence lower bound (ELBO) and the Kullback-Liebler (KL) divergence between the variational and true posterior distribution of the latent variable ***γ***_*j*_:

$$\log\text{Pr}\left({\hat{\gamma }}_{1j},{\hat{\gamma }}_{2j}|{\hat{R}}_{1j},{\hat{R}}_{2j};\theta \right)=L\left(q\right)+\mathrm{KL}\left(q\|p\right),$$ (8)

where

$$\begin{array}{cc} L\left(q\right) & =\int_{\gamma_{j}}q\left(\gamma_{j}\right)\log\frac{Pr\left({\hat{\gamma }}_{1j},{\hat{\gamma }}_{2j},\gamma_{j}|{\hat{R}}_{1j},{\hat{R}}_{2j};\theta \right)}{q\left(\gamma_{j}\right)}d\gamma_{j}\\ KL\left(q\|p\right) & =\int_{\gamma_{j}}q\left(\gamma_{j}\right)\log\frac{q\left(\gamma_{j}\right)}{p\left(\gamma_{j}|{\hat{\gamma }}_{1j},{\hat{\gamma }}_{2j},{\hat{R}}_{1j},{\hat{R}}_{2j};\theta \right)}d\gamma_{j}. \end{array}$$ (9)

We adopt the mean-field form of the variational posterior distribution

$$q\left(\gamma_{j}\right)=\overset{m_{j}}{\underset{k=1}{\prod }}q\left(\gamma_{jk}\right)$$ (10)

to speed up the computational process. The analytical form of the variational posterior distribution is obtained by minimizing the KL divergence, and the derived variational parameters are plugged back into the ELBO. The model parameters are then updated by setting the derivative of the ELBO with respect to the parameters equal to zero. By maximizing the ELBO with respect to the expanded parameter *τ*, we are able to further increase the ELBO and speed up the convergence process. Since the parameter-expanded model reduces to the original model when *τ* = 1, the original model can be recovered by incorporating *τ* into the model parameters, as outlined in the Supplementary Material.

### 2.4 Likelihood Ratio Test to Evaluate Expression-Trait Association

We perform a likelihood ratio test for expression-trait association:

$$H_{0}:\alpha_{j}=0\;\;H_{a}:\alpha_{j}\neq 0,$$ (11)

with the assumption of no horizontal pleiotropy. This is equivalent to testing

$$H_{0}:c_{j}\alpha_{j}=0\;\;H_{a}:c_{j}\alpha_{j}\neq 0,$$ (12)

since *c*_*j*_ ≠ 0. The test statistic for the *j*-th gene is

$$\Lambda_{j}=2\left(\log\text{Pr}\left({\hat{\gamma }}_{1j},{\hat{\gamma }}_{2j}|{\hat{R}}_{1j},{\hat{R}}_{2j};{\hat{\theta }}^{\text{ML}}\right)-\log\text{Pr}\left({\hat{\gamma }}_{1j},{\hat{\gamma }}_{2j}|{\hat{R}}_{1j},{\hat{R}}_{2j};{\hat{\theta }}_{0}^{\text{ML}}\right)\right),$$ (13)

where ${\hat{\theta }}_{0}^{\text{ML}}$ and ${\hat{\theta }}^{\text{ML}}$ are parameter estimates obtained by maximizing the marginal likelihood under $H_{0}$ and $H_{0}\cup H_{a}$, respectively. The test statistic asymptotically follows the $\chi_{\text{df}=1}^{2}$ under the null hypothesis (Van der Vaart, 2000), and the calculation of the marginal log-likelihood is detailed in the Supplementary Material. In practice, horizontal pleiotropy may be present, and the null hypothesis for CoMM-S^4^ becomes “there is no expression-trait effect and no horizontal pleitoropy.” As with other TWAS methods, horizontal pleiotropy could produce significant associations and inflation of test statistics (Gusev et al., 2016; Barbeira et al., 2018).

## 3 Results

### 3.1 Simulation Studies

In the simulation studies, we primarily focus on a) comparing the likelihood ratio test statistics from CoMM-S^4^ and CoMM-S^2^, b) assessing the type-I error of CoMM-S^4^ under the null hypothesis ($h_{T}^{2}=0$), and c) comparing the power of CoMM-S^4^, CoMM-S^2^ and S-PrediXcan.

#### 3.1.1 Simulation Settings

When comparing the test statistic and type-I error of CoMM-S^4^ with CoMM-S^2^, the sample sizes of the eQTL and GWAS datasets are *n*_1_ = 5, 000 and *n*_2_ = 5, 000 respectively. In the power comparison with CoMM-S^2^ and S-PrediXcan, the sample sizes are *n*_1_ = 500 and *n*_2_ = 10, 000 respectively. For all simulation scenarios, the sample size of the reference panels for the eQTL and GWAS datasets are *n*_3_ = 400 and *n*_4_ = 400 respectively.

A multivariate normal distribution with the covariance structure N(0,Σ(ρ)) is used to generate a prototype of the genotype matrix, where the parameter *ρ* ∈{0.2, 0.5, 0.8} determines the strength of correlations among the SNPs. Subsequently, minor allele frequencies are generated from the uniform distribution U(0.05,0.5). At each SNP position, the probability that an individual has 0, 1 or 2 minor alleles is calculated using the minor allele frequencies, assuming Hardy-Weinberg Equilibrium; individuals are assigned genotype values such that the desired genotype probabilities and minor allele frequencies are achieved.

We generate gene expression values according to **y**_*j*_ = **W**_1*j*_ ***γ***_*j*_ + **e**_1_, where $e_{1}∼N\left(0,\sigma_{e_{1}}^{2}I_{n_{1}}\right)$. The effect sizes *γ*_*jk*_ are generated from $N\left(0,\sigma_{\gamma_{j}}^{2}\right)$ with probability *π* and set to 0 with probability 1 − *π*, where *π* denotes the sparsity level and *k* indexes the genetic variants within a pre-defined window of gene *j*. To simulate distinct scenarios, we choose equally-spaced cellular heritability levels ($h_{C}^{2}$) of 0.01, 0.03, 0.05, 0.07, and 0.09, and sparsity levels of 0.1, 0.2, 0.3, 0.4, 0.5, and 1. Complex traits are generated according to **z** = *α*_*j*_ **W**_2*j*_ ***γ***_*j*_ + **e**_2_ and the number of *cis*-SNPs is set to 100. The trait level heritability ($h_{T}^{2}$) is set to 0 under the null hypothesis and 0.001, 0.002, and 0.003 under the alternative hypothesis.

The corresponding summary statistics were generated by applying a simple linear regression to the individual-level eQTL and GWAS datasets. Further details on the simulation procedure are in the Supplementary Material.

#### 3.1.2 Simulation Results

**FIGURE 1:**
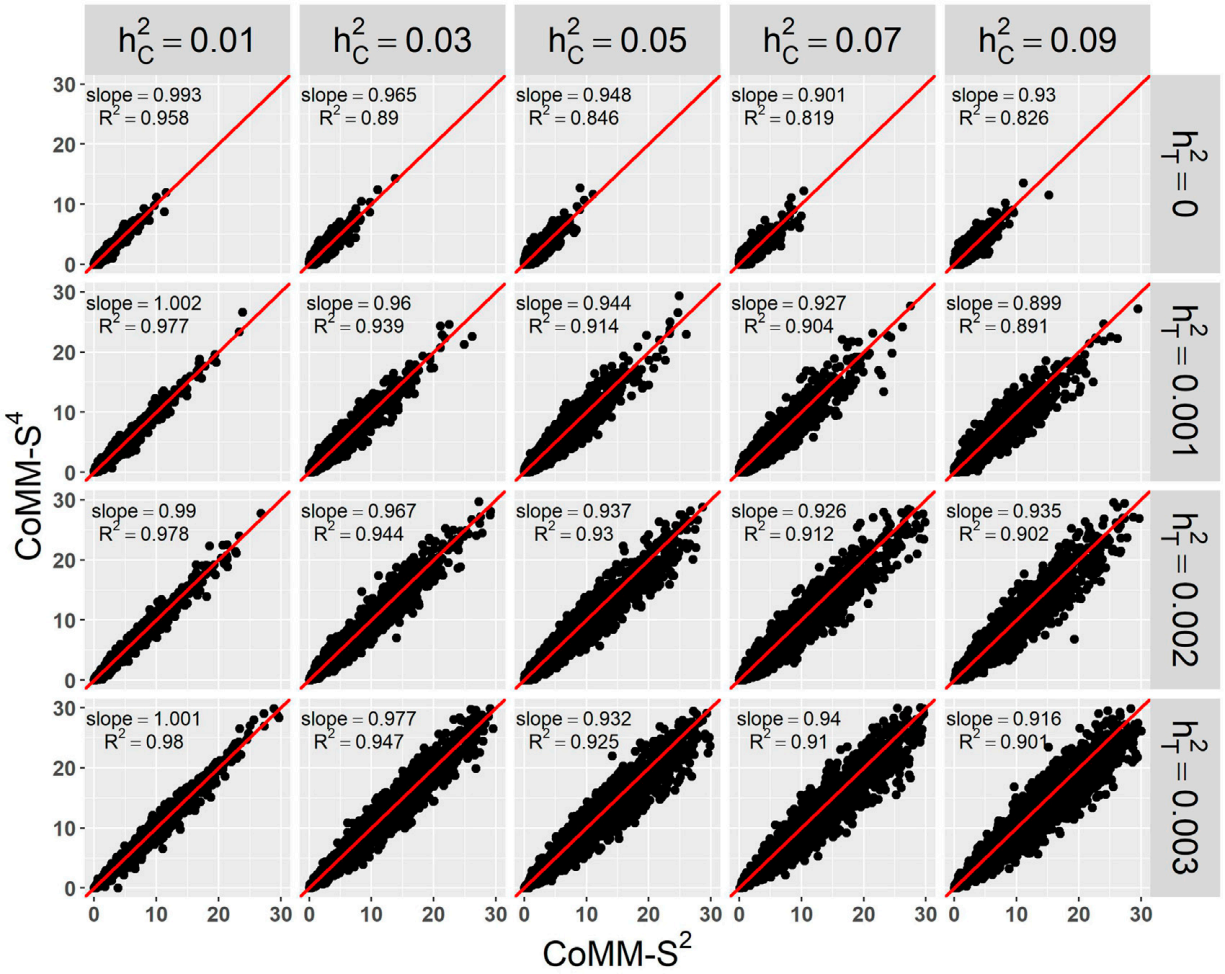
The scatter plot of CoMM-S^4^ vs. CoMM-S^2^, the model setting is *n*_1_ = 5,000, *n*_2_ = 5,000, *n*_3_ = 400, *n*_4_ = 400, *m*_*j*_ = 100, *ρ* = 0.5, *π* = 0.2, the number of replication is 2,000.

**FIGURE 2:**
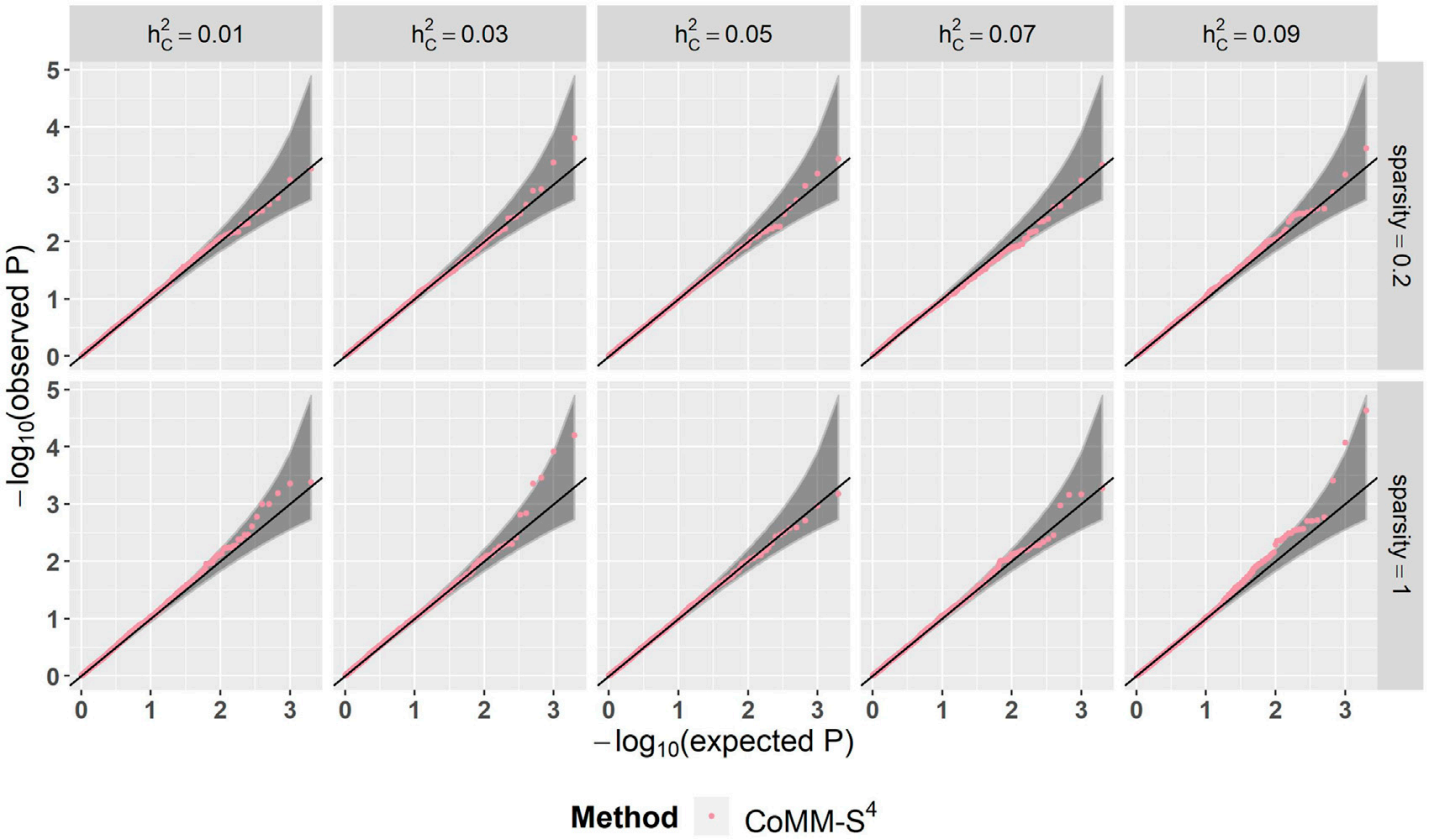
The QQ plot of CoMM-S^4^, the model setting is *n*_1_ = 5,000, *n*_2_ = 5,000, *n*_3_ = 400, *n*_4_ = 400, *ρ* = 0.5, the number of replication is 2,000.

There is a high concordance between the likelihood ratio test statistics from CoMM-S^4^ and CoMM-S^2^, which suggests that eQTL summary statistics can generally provide comparable power as individual-level data. In the scatter plot of CoMM-S^4^ and CoMM-S^2^ test statistics, the *R* ^2^ value is greater than 80% and the simple linear regression slope ranges from 0.88 to 1 (Figure 1 and Supplementay Figures S1–S6). Moreover, the QQ plots indicate that the observed *p*-values from CoMM-S^4^ are close to the expected *p*-values under the null hypothesis of no expression-trait association (Figure 2, Supplementary Figures S7–S9), indicating good type-I error control.

**FIGURE 3:**
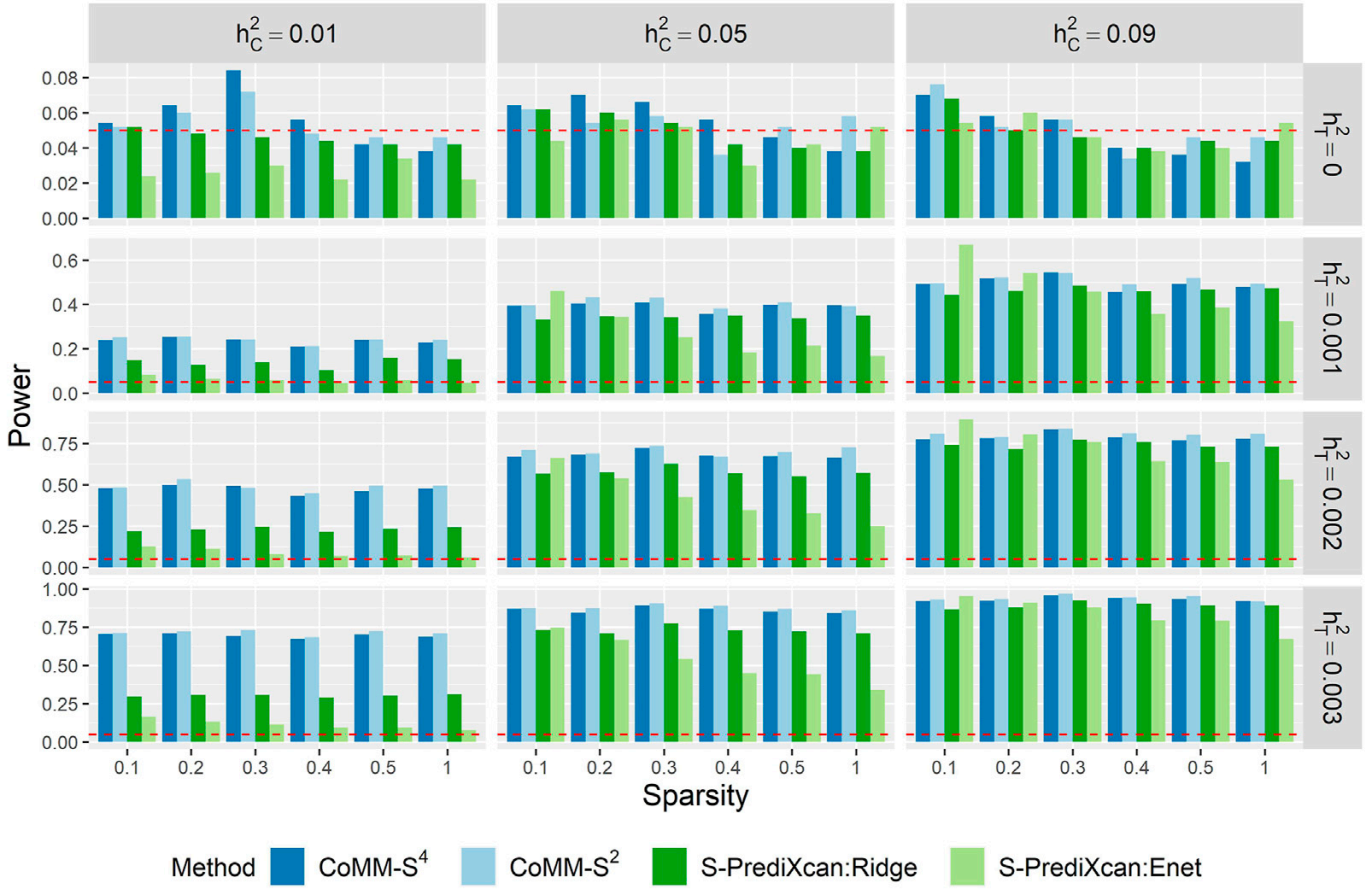
The empirical type I error ($h_{T}^{2}=0$) and power ($h_{T}^{2}>0$) of CoMM-S^4^, CoMM-S^2^, S-PrediXcan (ridge) and S-PrediXcan (elastic net) across 500 replications. The model setting is *n*_1_ = 500, *n*_2_ = 10,000, *n*_3_ = 400, *n*_4_ = 400, *ρ* = 0.5.

The power of CoMM-S^4^, CoMM-S^2^ and S-PrediXcan is also evaluated in the following scenarios: i) the eQTL and GWAS populations have the same LD structure, ii) the eQTL and GWAS populations have different LD structures, and iii) the eQTL and GWAS populations have different LD structures and different gene expression architectures, i.e. the set of *cis*-SNPs for the two populations only partially overlap (Figure 3, Supplementary Figures S10–S14; simulation details in Supplementary Material).

Across the scenarios considered, the greatest gains in power were observed when the cellular heritability is low ($h_{C}^{2}=0.01$) and the trait heritability is high ($h_{T}^{2}=0.003$). When the eQTL and GWAS samples are drawn from the same population, there is 71% power for CoMM-S^4^, compared with 30 and 16% power for S-PrediXcan (ridge) and S-PrediXcan (elastic net), respectively (sparsity = 0.1; Figure 3). When the eQTL and GWAS samples have distinct LD structures, there is 76% power for CoMM-S^4^, compared with 38 and 15% power for S-PrediXcan (ridge) and S-PrediXcan (elastic net), respectively (sparsity = 0.1; Figure S13). When the eQTL and GWAS samples have distinct LD structures and different gene expression architectures, there is 67% power for CoMM-S^4^, compared with 21 and 10% power for S-PrediXcan (ridge) and S-PrediXcan (elastic net), respectively (sparsity = 0.1; Supplementary Figure S14).

When the cellular heritability is large ($h_{C}^{2}=0.09$) and the gene expression architecture is the same in both the eQTL and GWAS datasets, the power of CoMM-S^4^ is comparable to S-PrediXcan (Figure 3; Supplementary Figures S10–S13). However, when the eQTL and GWAS samples have distinct LD structures and different gene expression architectures, CoMM-S^4^ shows some improvement in power over S-PrediXcan: there is 61% power for CoMM-S^4^, compared with 39 and 48% power for S-PrediXcan (ridge) and S-PrediXcan (elastic net), respectively ($h_{T}^{2}=0.003$, sparsity = 0.1; Supplementary Figure S14).

### 3.2 Real Data Analysis

#### 3.2.1 NFBC1966 Cohort

In the real data analysis, we apply CoMM-S^4^ to the NFBC1966 dataset (Sabatti et al., 2009). The NFBC1966 dataset contains phenotype data for the following ten traits: body mass index (BMI), systolic blood pressure (SysBP), diastolic blood pressure (DiaBP), high-density lipoprotein cholesterol (HDL-C), low-density lipoprotein cholesterol (LDL-C), triglycerides (TG), total cholesterol (TC), insulin levels, glucose levels and C-reactive protein (CRP). The summary statistics were generated by applying simple linear regression to individual-level NFBC1966 using plink (Purcell et al., 2007). Summary statistics of *cis*-eQTLs from eQTLGen Consortium (Võsa et al., 2018) were used. In addition, linkage disequilibrium for the eQTL and GWAS datasets was estimated using the 1,000 Genomes dataset (The 1000 Genomes Project Consortium, 2015) and 400 NFBC subsamples, respectively.

The genomic inflation factor is between 0.91 and 1.09, and the number of significant genes (*p*-value < 5 × 10^−6^) identified by CoMM-S^4^ is between 0 and 64 (Table 1). For the trait HDL, CoMM-S^4^ identified 61 genes, of which 20 are reported to be associated with HDL in NHGRI-EBI GWAS Catalog (Buniello et al., 2018). For the trait LDL, CoMM-S^4^ detected 64 genes, of which 13 are reported in GWAS Catalog. The corresponding QQ plots for these ten traits are illustrated in Supplementary Figure S15.

**TABLE 1:** The genomic inflation factor (GIF) and the number of associated genes (*p*-value <5 × 10^−6^) found by CoMM-S^4^ for the ten NFBC traits. The number within the parentheses is the number of associated genes reported in the NHGRI-EBI GWAS Catalog (Buniello et al., 2018).

	GIF	No. of associated genes (reported in GWAS Catalog)
CRP	0.94	25 (5)
Glucose	0.99	4 (1)
Insulin	0.86	1 (0)
TC	1.06	26 (4)
HDL	1.09	61 (20)
LDL	1.09	64 (13)
TG	1.06	2 (0)
BMI	0.98	3 (1)
SysBP	1.05	0 (0)
DiaBP	0.91	0 (0)

#### 3.2.2 Biobank Japan

We apply CoMM-S^4^ to GWAS summary statistics from Biobank Japan (BBJ) (Ishigaki et al., 2020). We considered two autoimmune traits (Graves’ disease, rheumatoid arthritis), four cardiovascular traits (cerebral aneurysm, congestive heart failure, ischemic stroke, peripheral artery disease), two infection-related traits (chronic hepatitis B, chronic hepatitis C) and osteoporosis. The TWAS analysis is performed using whole-blood *cis*-eQTL summary statistics from two studies, eQTLGen (Võsa et al., 2018) and GTEx (v8) (The GTEx Consortium, 2020), to assess the robustness of TWAS results to choice of eQTL dataset. The GTEx and eQTLGen datasets contain association results for 19,599 and 19,176 genes respectively, of which 16,692 genes are in common. Linkage disequilibrium corresponding to the GWAS and eQTL datasets were estimated using Japanese and European samples from the 1,000 Genomes Project (The 1000 Genomes Project Consortium, 2015), respectively. As population differences in eQTL architecture may reduce gene expression imputation accuracy for the GWAS samples, it is preferable for the eQTL and GWAS data to be collected from the same population (Keys et al., 2020). However, the availability of highly-powered eQTL studies may be limited for the population of interest. Moreover, populations that are closely related still provide good power to detect associations between gene expression and trait (Keys et al., 2020), and the relatively high concordance rate (68.8%) of *cis*-regulation in European and Japanese eQTL studies (Narahara et al., 2014) suggest that European eQTL studies could serve as a reasonable proxy.

TWAS was performed to find genetic loci that may be associated with the traits of interest. For traits where TWAS identified more than 100 statistically significant genes, we further carried out an enrichment analysis based on gene ontology (GO) terms using Enrichr (Chen et al., 2013). The genomic inflation factor is between 1.06 and 1.30 when eQTL summary statistics were obtained from eQTLGen, and between 0.87 and 1.26 when eQTL summary statistics were obtained from GTEx (v8) whole blood (Table 2). The number of identified genes (*p*-value < 5 × 10^−6^) ranged from 2 to 450, and there is a high degree of overlap between the genes identified in the two analyses (Table 2), indicating robustness to eQTL dataset choice. Moreover, around half or more of the genes identified by CoMM-S^4^ have not been previously reported as significant in the GWAS Catalog or the Biobank GWAS analysis (Table 2).

**TABLE 2:** The genomic inflation factor and number of associated genes (*p*-value <5 × 10^−6^) for 9 traits in the Biobank Japan dataset. Two eQTL datasets were used: eQTLGen and GTEx. In parentheses are the number of associated genes that are also present in the other eQTL dataset’s gene set. The last column shows the number of associated genes that are common to both the eQTLGen and GTEx analyses; in parentheses are the number of associated genes that are statistically significant in the GWAS analysis (*p*-value <5 × 10^−8^), and the number of associated genes reported in the GWAS Catalog.

	eQTLGen		GTEx		eQTLGen and GTEx
	GIF	No. associated genes (No. in GTEx)	GIF	No. associated genes (No. in eQTLGen)	No. common associated genes (sig. in BBJ GWAS; reported in GWAS Catalog)
Graves’ disease	1.17	283 (247)	1.09	454 (364)	245 (125; 7)
Rheumatoid arthritis	1.30	266 (230)	1.26	402 (323)	220 (134; 22)
Chronic hepatitis B	1.06	148 (133)	0.87	211 (172)	132 (70; 6)
Chronic hepatitis C	1.09	73 (66)	1.00	163 (145)	64 (4; 1)
Ischemic stroke	1.25	23 (21)	1.24	60 (56)	19 (3; 3)
Congestive heart failure	1.18	4 (2)	1.13	10 (9)	1 (0; 0)
Peripheral artery disease	1.13	13 (10)	0.99	45 (37)	7 (0; 0)
Cerebral aneurysm	1.11	4 (4)	0.99	6 (6)	2 (0; 0)
Osteoporosis	1.07	2 (2)	0.93	7 (6)	1 (0; 0)

The TWAS results recapitulate known or proposed biological mechanisms that give rise to the studied traits. GWAS and animal model studies have implicated MHC molecules, interferon-gamma signalling and apoptosis in the development of Graves’ disease (Morshed and Davies, 2015; Okada et al., 2015; Smith and Hegedüs, 2016), rheumatoid arthritis (Castañeda-Delgado et al., 2017; Okada et al., 2019), and chronic hepatitis B infection (Ebert et al., 2015; Zhu et al., 2016). Pathway enrichment analyses recapitulate these findings. For Graves’ disease, 143 of the 245 associated genes (TWAS *p*-value < 5 × 10^−8^) are involved in GO biological processes, and the 23 significantly enriched processes (FDR <0.05, Supplementary Table S3) include interferon-gamma-mediated signaling pathway (*p* = 6.86 × 10^−10^), as well as antigen processing and presentation of peptide antigen via MHC class I (*p* = 3.14 × 10^−8^) and via MHC class II (*p* = 2.76 × 10^−6^). For rheumatoid arthritis, 137 of the 220 associated genes are involved in GO biological processes, and the 25 significantly enriched processes (Supplementary Table S4) include interferon-gamma-mediated signaling pathway (*p* = 1.20 × 10^−11^), and antigen processing and presentation of exogenous peptide antigen via MHC class II (*p* = 4.21 × 10^−11^). For chronic hepatitis B, 91 of the 132 associated genes are involved in GO biological processes, and the 32 significantly enriched processes (Supplementary Table S5) include antigen processing and presentation of exogenous peptide antigen via MHC class II (*p* = 2.28 × 10^−7^) and positive regulation of apoptotic cell clearance (*p* = 9.21 × 10^−6^).

Moreover, CoMM-S^4^ is able to identify novel susceptibility loci by aggregating the contributions of SNPs with smaller effect sizes. A comparison of the GWAS results with CoMM-S^4^ results based on the highly-powered eQTLGen study shows that the TWAS signal is larger than the GWAS signal at chr17q12 for congestive heart failure (CHF), chr17p13.1 for peripheral artery disease (PAD), chr17q21.31 for ischemic stroke, and chr6q22.33 for osteoporosis (Supplementary Figures S17–S25). Plausible mechanisms can be identified for genes at these loci, which may serve as a stepping stone for further investigation. For CHF, the second largest signal at chr17q12 corresponds to *FBXL20* (*p* = 1.33 × 10^−5^), which negatively regulates autophagy (Mathiassen and Cecconi, 2017). Reduced autophagy contributes to accelerated cardiac ageing and heart failure (Nishida et al., 2009; Abdellatif et al., 2018; Dong et al., 2019), and may serve as a link between *FBXL20* and CHF. For PAD, the second largest signal at chr17p13.1 corresponds to *GABARAP* (*p* = 8.63 × 10^−8^), which is involved in autophagy initiation and autophagosome-lysosome fusion (Schaaf et al., 2016). Impaired autophagy aggravates atherosclerosis (De Meyer et al., 2015), and may serve as a link between *GABARAP* and PAD.

For ischemic stroke, the TWAS signal is larger than the GWAS signal at chr17q21.31. The top association corresponds to *HEXIM1* (*p* = 1.07 × 10^−6^), which modulates hypoxia-inducible factor-1 alpha and vascular endothelial growth factor (Ogba et al., 2010; Ketchart et al., 2013), angiogenic factors which may influence stroke risk by mediating neovascularization in atherosclerotic lesions, potentially precipitating thrombi that obstruct blood flow to the brain (Bentzon et al., 2014; Chistiakov et al., 2015; Camaré et al., 2017). For osteoporosis, the TWAS signal is larger than the GWAS signal at chr6q22.33. The top association corresponds to *RNF146* (*p* = 1.05 × 10^−8^), which was shown to promote osteoblast development while antagonizing osteoclast differentiation in mice (Matsumoto et al., 2017). Notably, none of the genes described above are reported as significant in the GWAS Catalog or the Biobank Japan GWAS analysis, thus highlighting the potential utility of applying CoMM-S^4^ to identify relevant genes.

On the other hand, the TWAS results are limited by the data availability in the eQTL dataset. Although the TWAS results recapitulate most GWAS results, the Manhattan plots also show some GWAS signals without corresponding TWAS signals (Supplementary Figures S17–S25), in part due to the relative sparsity of genes in the eQTL dataset. A further limitation is that TWAS provide information about association, rather than causality. In the present analysis, *TMEM184C* and *PRMT10* showed significant association with cerebral aneurysm. However, a previous report has indicated that these are not the causal genes. Instead, the likely causal gene is *EDNRA*, which is in the same locus as *TMEM184C* and *PRMT10* and regulates response to hemodynamic stress (Low et al., 2012). As *EDNRA* is not present in any of the eQTL datasets, it could not be evaluated in this analysis.

In addition, we compare the CoMM-S^4^ results with S-PrediXcan (elastic net) results for the 9 Biobank Japan traits. For S-PrediXcan, gene expression prediction weights for GTEx (v8) whole blood were obtained from the elastic net model in PredictDB (http://predictdb.org/), and the covariance matrix used to calculate the test statistics is based on Japanese samples from the 1,000 Genomes Project. To allow for fair comparison, we consider only genes that are common to both the CoMM-S^4^ and S-PrediXcan analyses. Compared with S-PrediXcan (elastic net), CoMM-S^4^ identifies a similar number of statistically significant genes for 5 Biobank Japan traits (cerebral aneurysm, congestive heart failure, ischemic stroke, peripheral artery disease, and osteoporosis), and more statistically significant genes for 4 Biobank Japan traits (Graves’ disease, rheumatoid arthritis, chronic hepatitis C, and chronic hepatitis B) (Supplementary Table S2). The tail behaviour in the QQ plots indicate that the *p*-values tend to be smaller for statistically significant genes (Supplementary Figure S16). The higher number of identified genes in the Biobank Japan traits is consistent with the higher power demonstrated in simulations.

## 4 Discussion

In this article, we have developed a collaborative mixed model using both summary statistics from eQTL and GWAS to examine the expression-trait associations in transcriptome-wide association studies. We compared the performance between CoMM-S^4^ and CoMM-S^2^, and simulation results demonstrate that CoMM-S^4^ performs as well as CoMM-S^2^ even though the former uses only summary-level data. Moreover, our analysis of the NFBC1966 cohort has suggested novel susceptibility loci for glucose levels, insulin levels, C-reactive protein, BMI and lipid traits. Our analysis of Biobank Japan traits has similarly suggested novel susceptibility loci for congestive heart failure, ischemic stroke, peripheral artery disease and osteoporosis, and has also recapitulated known and putative mechanisms for Graves’ disease, rheumatoid arthritis, chronic hepatitis B and chronic hepatitis C.

CoMM-S^4^ has several advantages over CoMM-S^2^ and S-PrediXcan. Compared to stage-wise methods like S-PrediXcan, CoMM-S^4^ accounts for imputation uncertainty, which makes it statistically more powerful in identifying expression-trait associations. Moreover, CoMM-S^4^ requires only summary-level data (z-scores) from eQTL studies, instead of individual-level data. This allows us to make use of eQTL large-scale studies and meta-analyses where individual-level data may be unavailable.

On the other hand, likelihood-ratio tests are less computationally efficient than score-based tests; the relationship between these tests in the context of individual-level data (CoMM and SKAT, respectively) are discussed in detail in (Yang et al., 2018). To reduce the computational time of CoMM-S^4^, we have estimated the parameters using variational inference and parameter expansion. Finally, CoMM-S^4^, like S-PrediXcan, is not able to distinguish between causal relationship and horizontal pleiotropy. In practice, we can first perform a TWAS to identify regions that show association with the trait of interest, and then apply Mendelian randomization analysis to draw causal conclusions.

## Data Availability

Publicly available datasets were analyzed in this study. Biobank Japan GWAS summary statistics were obtained from http://jenger.riken.jp/en/result; eQTLGen summary statistics were obtained from https://www.eqtlgen.org/; GTEx eQTL summary statistics were obtained from https://www.ebi.ac.uk/eqtl/Studies/.
