# Supplementary material for: CoMM-S4: A Collaborative Mixed Model Using Summary-Level eQTL and GWAS Datasets in Transcriptome-Wide Association Studies
Source: Front Genet. 2021 Sep 20;12:704538. doi: 10.3389/fgene.2021.704538 (PMC8488198; doi:10.3389/fgene.2021.704538)
Supplement: Supplementary file 1 [file DataSheet1.PDF]

Supplementary Material

1 METHOD

1.1 Notation

The individual-level eQTL dataset for n_1 samples is denoted by $\{\mathbf{Y}, \mathbf{W}_1\}$, where $\mathbf{Y}$ is the gene expression matrix for g genes and $\mathbf{W}_1$ is the genotype matrix matrix for m single nucleotide polymorphism (SNP) positions. We construct separate models for each gene. For the j-th gene, let $\mathbf{y}_j$ denote the gene expression vector, and $\mathbf{W}_{1j} \in \mathbb{R}^{n_1 \times m_j}$ denote the centered genotype matrix for the m_j SNPs within a pre-defined distance from the gene. In addition, let the individual-level GWAS dataset for n_2 samples be denoted by $\{\mathbf{z}, \mathbf{W}_2\}$, where $\mathbf{z}$ is the phenotype vector and $\mathbf{W}_2$ is the genotype matrix. Similarly, for the j-th gene, $\mathbf{W}_{2j} \in \mathbb{R}^{n_2 \times m_j}$ denotes the centered genotype matrix for the m_j SNPs within a pre-defined distance from the gene.

We have the summary statistics $\mathcal{D}_1 = \{\widehat{\boldsymbol{\gamma}}_1\}$ from the analysis of gene expression-genetic variant pairs in the eQTL dataset, where $\widehat{\boldsymbol{\gamma}}_1$ is the ratio of effect size to the corresponding standard deviation (z-score). We also have the summary statistics $\mathcal{D}_2 = \{\widehat{\boldsymbol{\gamma}}_2\}$ from single-variate analysis in the GWAS dataset. We denote the eQTL z-scores for the j-th gene by $\widehat{\boldsymbol{\gamma}}_{1j} \in \mathbb{R}^{m_j}$, and the GWAS z-scores by $\widehat{\boldsymbol{\gamma}}_{2j} \in \mathbb{R}^{m_j}$. To model the linkage disequilibrium (LD) in the eQTL and GWAS datasets, we require the corresponding sample correlation matrices, denoted as $\widehat{\mathbf{R}}_{1j} \in \mathbb{R}^{m_j \times m_j}$ and $\widehat{\mathbf{R}}_{2j} \in \mathbb{R}^{m_j \times m_j}$ $(j = 1, \ldots, g)$, respectively.

1.2 The basic model

The relationship between the j-th gene expression $\mathbf{y}_j$ and genotype $\mathbf{W}_{1j}$ is modelled as

$$\mathbf{y}_j = \mathbf{W}_{1j}\boldsymbol{\beta}_{1j} + \mathbf{e}_1, \tag{S1}$$

where $\boldsymbol{\beta}_{1j} = [\beta_{1j,1}, \ldots, \beta_{1j,m_j}]^T$ is an m_j-vector of effect sizes, and $\mathbf{e}_1 \sim \mathcal{N}(\mathbf{0}, \sigma^2_{e_1}\mathbf{I})$ is an n_1-vector of independent noise. Similarly, the relationship between trait $\mathbf{z}$ and genotype $\mathbf{W}_{2j}$ is modelled as

$$\mathbf{z} = \mathbf{W}_{2j}\boldsymbol{\beta}_{2j} + \mathbf{e}_2, \tag{S2}$$

where $\boldsymbol{\beta}_{2j} = [\beta_{2j,1}, \ldots, \beta_{2j,m_j}]^T$ is an m_j-vector of effect sizes, and $\mathbf{e}_2 \sim \mathcal{N}(\mathbf{0}, \sigma^2_{e_2}\mathbf{I})$ is an n_2-vector of independent noise. We further model the GWAS effect size as $\boldsymbol{\beta}_{2j} = \alpha_j\boldsymbol{\beta}_{1j}$, where α_j can be interpreted as the effect of gene expression on phenotype. To perform a likelihood ratio test for the null hypothesis $\alpha_j = 0$, we first derive the form of the log-likelihood and develop an efficient algorithm to estimate its parameters.

Let $\widehat{\gamma}_{1j}$ and $\widehat{\gamma}_{2j}$ denote the z-score vectors for the eQTL and GWAS data, respectively. Let $\widehat{\mathrm{s}}_{1j}$ and $\widehat{\mathrm{s}}_{2j}$ denote the standard errors of the effect size estimators, $\widehat{\boldsymbol{\beta}_j}$ and $\widehat{\alpha_j\boldsymbol{\beta}_j}$, in the eQTL and GWAS analyses respectively. We assume that the z-scores are available. Based on the "Regression with Summary Statistics" (RSS) likelihood (Zhu and Stephens, 2017), the distribution of the eQTL z-scores $\widehat{\gamma}_{1j}$ can be approximated by

$$\widehat{\gamma}_{1j} \mid \boldsymbol{\gamma}_j, \widehat{\mathbf{R}}_{1j} \sim \mathcal{N}(\widehat{\mathbf{R}}_{1j}\boldsymbol{\gamma}_j, \widehat{\mathbf{R}}_{1j}), \tag{S3}$$

where $\boldsymbol{\gamma}_j = \widehat{\mathbf{S}}_{1j}^{-1}\boldsymbol{\beta}_j$ and $\widehat{\mathbf{S}}_{ij} = \mathrm{diag}(\widehat{\mathrm{s}}_{ij})$ $(i = 1, 2)$. Similarly, the distribution of the GWAS z-scores $\widehat{\gamma}_{2j}$ can be approximated by

$$\widehat{\gamma}_{2j} \mid \boldsymbol{\gamma}_j, \widehat{\mathbf{R}}_{2j} \sim \mathcal{N}(\alpha_j c_j \widehat{\mathbf{R}}_{2j}\boldsymbol{\gamma}_j, \widehat{\mathbf{R}}_{2j}), \tag{S4}$$

where $c_j \approx \frac{\hat{\sigma}_{yj}}{\hat{\sigma}_z}\sqrt{\frac{n_2}{n_1}}$ when the summary statistics are generated using simple linear regression, $\hat{\sigma}_{yj}$ is the sample standard deviation for the expression of gene j, and $\hat{\sigma}_z$ is the sample standard deviation of the trait. The mean in S4 arises because the RSS mean is $\widehat{\mathbf{R}}_{2j}\widehat{\mathbf{S}}_{2j}^{-1}(\alpha_j\boldsymbol{\beta}_j) = \alpha_j\widehat{\mathbf{R}}_{2j}\widehat{\mathbf{S}}_{2j}^{-1}\widehat{\mathbf{S}}_{1j}\boldsymbol{\gamma}_j$ $= \alpha_j\widehat{\mathbf{R}}_{2j}\Big(\frac{\hat{\sigma}_z}{\sqrt{n_2}}\mathrm{diag}\big(\frac{1}{\hat{\sigma}_{x,2j,1}}, \ldots, \frac{1}{\hat{\sigma}_{x,2j,m_j}}\big)\Big)^{-1}\Big(\frac{\hat{\sigma}_{yj}}{\sqrt{n_1}}\mathrm{diag}\big(\frac{1}{\hat{\sigma}_{x,1j,1}}, \ldots, \frac{1}{\hat{\sigma}_{x,1j,m_j}}\big)\Big)\boldsymbol{\gamma}_j$, where $\hat{\sigma}_{x,1j,k}$ and $\hat{\sigma}_{x,2j,k}$ are the sample standard deviations at SNP k $(k = 1, \ldots, m_j)$ in the eQTL and GWAS data, respectively. Furthermore, we include a Gaussian prior for $\boldsymbol{\gamma}_j$,

$$\boldsymbol{\gamma}_j \sim \mathcal{N}(0, \sigma^2_{\gamma_j}\mathbf{I}_{m_j}). \tag{S5}$$

The complete-data likelihood can be written as

$$\mathrm{Pr}(\widehat{\gamma}_{1j}, \widehat{\gamma}_{2j}, \boldsymbol{\gamma}_j \mid \widehat{\mathbf{R}}_{1j}, \widehat{\mathbf{R}}_{2j}; \boldsymbol{\theta}) = \prod_{i=1}^{2}\mathrm{Pr}(\widehat{\gamma}_{ij} \mid \boldsymbol{\gamma}_j, \widehat{\mathbf{R}}_{ij})\mathrm{Pr}(\boldsymbol{\gamma}_j), \tag{S6}$$

where $\boldsymbol{\theta} = \{\sigma^2_{\gamma_j}, \alpha_j\}$ is the collection of parameters. By integrating out the latent variable $\boldsymbol{\gamma}_j$, we obtain the marginal likelihood

$$\mathrm{Pr}(\widehat{\gamma}_{1j}, \widehat{\gamma}_{2j} \mid \widehat{\mathbf{R}}_{1j}, \widehat{\mathbf{R}}_{2j}; \boldsymbol{\theta}) = \int_{\boldsymbol{\gamma}_j}\mathrm{Pr}(\widehat{\gamma}_{1j}, \widehat{\gamma}_{2j}, \boldsymbol{\gamma}_j \mid \widehat{\mathbf{R}}_{1j}, \widehat{\mathbf{R}}_{2j}; \boldsymbol{\theta})\, d\boldsymbol{\gamma}_j. \tag{S7}$$

We are interested in the parameter α_j, which is the effect of gene expression on phenotype. The accuracy of the above distributional approximations depend on the sample size of the eQTL and GWAS datasets, as well as the number of SNPs/genes associated with the gene expression/phenotype. The larger the sample size and the higher the degree of polygenecity, the greater the estimation accuracy.

1.3 Parameter expansion

An efficient algorithm is needed to estimate the parameters of the model. Although the EM algorithm is widely used and has a highly stable performance, it requires inverting the matrix $\widehat{\mathbf{R}}_{1j}$ and $\widehat{\mathbf{R}}_{2j}$ in each iteration, which adds to the computational burden. Hence, we adopt the Variational Bayes Expectation-mMximization (VBEM) algorithm instead to speed up the computational process. Furthermore, we use parameter expansion to further improve the speed of VBEM algorithm. The parameter-expanded model is

$$\widehat{\gamma}_{1j} \mid \boldsymbol{\gamma}_j, \widehat{\mathbf{R}}_{1j} \sim \mathcal{N}(\tau \widehat{\mathbf{R}}_{1j} \boldsymbol{\gamma}_j, \widehat{\mathbf{R}}_{1j}), \tag{S8}$$

where the $\tau \in \mathbb{R}$ is the expanded parameter. The distribution of $\widehat{\gamma}_{2j}$ and the prior for the $\boldsymbol{\gamma}_j$ remain the same, but the model parameters become $\boldsymbol{\theta} = \{\sigma^2_{\gamma_j}, \alpha_j, \tau\}$.

Here, we briefly sketch the basic idea of the parameter expansion Variational Bayes Expectation-Maximization (PX-VBEM). Given the variational posterior distribution $q(\boldsymbol{\gamma}_j)$, the marginal log-likelihood can be decomposed into two components, the evidence lower bound (ELBO) and the Kullback-Liebler divergence between the variational and true posterior distribution of the latent variable

$$\log \Pr(\widehat{\gamma}_{1j}, \widehat{\gamma}_{2j} \mid \widehat{\mathbf{R}}_{1j}, \widehat{\mathbf{R}}_{2j}; \boldsymbol{\theta}) = \mathcal{L}(q) + \mathbb{KL}(q||p), \tag{S9}$$

where

$$\begin{aligned} \mathcal{L}(q) &= \int_{\boldsymbol{\gamma}_j} q(\boldsymbol{\gamma}_j) \log \frac{\Pr(\widehat{\gamma}_{1j}, \widehat{\gamma}_{2j}, \boldsymbol{\gamma}_j \mid \widehat{\mathbf{R}}_{1j}, \widehat{\mathbf{R}}_{2j}; \boldsymbol{\theta})}{q(\boldsymbol{\gamma}_j)} d\boldsymbol{\gamma}_j \\ \mathbb{KL}(q||p) &= \int_{\boldsymbol{\gamma}_j} q(\boldsymbol{\gamma}_j) \log \frac{q(\boldsymbol{\gamma}_j)}{p(\boldsymbol{\gamma}_j \mid \widehat{\gamma}_{1j}, \widehat{\gamma}_{2j}, \widehat{\mathbf{R}}_{1j}, \widehat{\mathbf{R}}_{2j}; \boldsymbol{\theta})} d\boldsymbol{\gamma}_j. \end{aligned} \tag{S10}$$

To efficiently evaluate the lower bound, we adopt the mean field form (Opper and Saad, 2001) of $q(\boldsymbol{\gamma}_j)$

$$q(\boldsymbol{\gamma}_j) = \prod_{k=1}^{m_j} q(\gamma_{jk}). \tag{S11}$$

The analytical form of the variational posterior distribution using the formula (10.9) in Bishop (Bishop, 2006), and the details are in the Section 1.4.1. After obtaining the variational posterior distribution, we can evaluate the ELBO (Section 1.4.2). Finally, we derive the updating formulas of parameters by setting the derivative of ELBO with respect to the parameters equal to zero (Section 1.4.3).

1.4 Variational PX-EM algorithm

1.4.1 Variational distribution of latent variables

The complete-data log-likelihood is

$$\begin{aligned}
&\log \Pr(\widehat{\boldsymbol{\gamma}}_{1j}, \widehat{\boldsymbol{\gamma}}_{2j}, \boldsymbol{\gamma}_j \mid \widehat{\mathbf{R}}_{1j}, \widehat{\mathbf{R}}_{2j}; \boldsymbol{\theta}) \\
=& -\frac{1}{2}\log|\widehat{\mathbf{R}}_{1j}| - \frac{1}{2}\log|\widehat{\mathbf{R}}_{2j}| \\
&- \frac{1}{2}(\widehat{\boldsymbol{\gamma}}_{1j} - \tau\widehat{\mathbf{R}}_{1j}\boldsymbol{\gamma}_j)^\top \widehat{\mathbf{R}}_{1j}^{-1}(\widehat{\boldsymbol{\gamma}}_{1j} - \tau\widehat{\mathbf{R}}_{1j}\boldsymbol{\gamma}_j) \\
&- \frac{1}{2}(\widehat{\boldsymbol{\gamma}}_{2j} - \alpha_j c_j\widehat{\mathbf{R}}_{2j}\boldsymbol{\gamma}_j)^\top \widehat{\mathbf{R}}_{2j}^{-1}(\widehat{\boldsymbol{\gamma}}_{2j} - \alpha_j c_j\widehat{\mathbf{R}}_{2j}\boldsymbol{\gamma}_j) \\
&- \frac{m_j}{2}\log(2\pi\sigma^2_{\gamma_j}) - \frac{||\boldsymbol{\gamma}_j||^2}{2\sigma^2_{\gamma_j}}
\end{aligned} \tag{S12}$$

and the terms with γ_{jk} has the following quadratic form

$$\begin{aligned}
&-\frac{1}{2}\left(\tau^2 R_{1jkk} + \alpha_j^2 c_j^2 R_{2jkk} + \frac{1}{\sigma^2_{\gamma_j}}\right)\gamma_{jk}^2 \\
&+ \left(\tau\widehat{\gamma}_{1jk} - \tau^2\sum_{i\neq k}\gamma_{ji}R_{1jki} + \alpha_j c_j \widehat{\gamma}_{2jk} - \alpha_j^2 c_j^2 \sum_{i\neq k}\gamma_{ji}R_{2jki}\right)\gamma_{jk}.
\end{aligned} \tag{S13}$$

The analytical form of the variational posterior distribution is obtained using formula (10.9) in (Bishop, 2006). The log of the probability density function of γ_{ji}, $q(\gamma_{ji})$ $(i = 1, \ldots, {}_j)$, is

$$\begin{aligned}
\log q(\gamma_{jk}) =& -\frac{1}{2}\left(\tau^2 R_{1jkk} + \alpha_j^2 c_j^2 R_{2jkk} + \frac{1}{\sigma^2_{\gamma_j}}\right)\gamma_{jk}^2 \\
&+ \left(\tau\widehat{\gamma}_{1jk} - \tau^2\sum_{i\neq k}\mathbb{E}[\gamma_{ji}]R_{1jki} + \alpha_j c_j \widehat{\gamma}_{2jk} - \alpha_j^2 c_j^2 \sum_{i\neq k}\mathbb{E}[\gamma_{ji}]R_{2jki}\right)\gamma_{jk}.
\end{aligned} \tag{S14}$$

Hence, the variational distribution of γ_{jk} is a Gaussian distribution $\mathcal{N}(u_{jk}, v_{jk}^2)$, with mean u_{jk} and variance v_{jk}^2 defined as

$$\begin{aligned}
v_{jk}^2 &= \frac{1}{\tau^2 R_{1jkk} + \alpha_j^2 c_j^2 R_{2jkk} + \frac{1}{\sigma^2_{\gamma_j}}}, \\
u_{jk} &= \frac{\tau\widehat{\gamma}_{1jk} - \tau^2\sum_{i\neq k}\mathbb{E}[\gamma_{ji}]R_{1jki} + \alpha_j c_j\widehat{\gamma}_{2jk} - \alpha_j^2 c_j^2\sum_{i\neq k}\mathbb{E}[\gamma_{ji}]R_{2jki}}{\tau^2 R_{1jkk} + \alpha_j^2 c_j^2 R_{2jkk} + \frac{1}{\sigma^2_{\gamma_j}}}.
\end{aligned} \tag{S15}$$

1.4.2 Evidence lower bound (ELBO)

The evidence lower bound (ELBO) $\mathcal{L}(q)$ is

$$\mathcal{L}(q) = \mathbb{E}_q[\log \Pr(\widehat{\boldsymbol{\gamma}}_{1j}, \widehat{\boldsymbol{\gamma}}_{2j}, \boldsymbol{\gamma}_j \mid \widehat{\mathbf{R}}_{1j}, \widehat{\mathbf{R}}_{2j}; \boldsymbol{\theta})] - \mathbb{E}_q[\log q(\boldsymbol{\gamma}_j)]. \tag{S16}$$

The first term of ELBO is

$$\begin{aligned}
&\mathbb{E}_q[\log \Pr(\widehat{\boldsymbol{\gamma}}_{1j}, \widehat{\boldsymbol{\gamma}}_{2j}, \boldsymbol{\gamma}_j \mid \widehat{\mathbf{R}}_{1j}, \widehat{\mathbf{R}}_{2j}; \boldsymbol{\theta})] \\
=& -\frac{1}{2}\log|\widehat{\mathbf{R}}_{1j}| - \frac{1}{2}\log|\widehat{\mathbf{R}}_{2j}| \\
&-\frac{1}{2}\mathbb{E}_q[(\widehat{\boldsymbol{\gamma}}_{1j} - \tau\widehat{\mathbf{R}}_{1j}\boldsymbol{\gamma}_j)^\top(\widehat{\mathbf{R}}_{1j})^{-1}(\widehat{\boldsymbol{\gamma}}_{1j} - \tau\widehat{\mathbf{R}}_{1j}\boldsymbol{\gamma}_j)] \\
&-\frac{1}{2}\mathbb{E}_q[(\widehat{\boldsymbol{\gamma}}_{2j} - \alpha_j c_j\widehat{\mathbf{R}}_{2j}\boldsymbol{\gamma}_j)^\top(\widehat{\mathbf{R}}_{2j})^{-1}(\widehat{\boldsymbol{\gamma}}_{2j} - \alpha_j c_j\widehat{\mathbf{R}}_{2j}\boldsymbol{\gamma}_j)] \\
&-\frac{m_j}{2}\log(2\pi\sigma^2_{\gamma_j}) - \frac{\mathbb{E}_q||\boldsymbol{\gamma}_j||^2}{2\sigma^2_{\gamma_j}}.
\end{aligned} \tag{S17}$$

Since

$$\begin{aligned}
\mathbb{E}_q[\boldsymbol{\gamma}_j] &= \mathbf{u}_j, \\
\mathbb{E}_q\|\boldsymbol{\gamma}_j\|^2 &= \mathbf{u}_j^\top\mathbf{u}_j + \mathbf{v}_j^\top\mathbf{v}_j, \\
\mathbb{E}_q[\boldsymbol{\gamma}_j^\top\widehat{\mathbf{R}}_{kj}\boldsymbol{\gamma}_j] &= \mathbf{u}_j^\top\widehat{\mathbf{R}}_{kj}\mathbf{u}_j + (\mathbf{v}_j \odot \mathbf{v}_j)^\top\text{diag}(\widehat{\mathbf{R}}_{kj}), (k = 1, 2)
\end{aligned} \tag{S18}$$

where the symbol $\odot$ denotes the element-wise multiplication, the required expectations can be expressed in terms of the parameters of the variational distribution:

$$\begin{aligned}
&\mathbb{E}_q[(\widehat{\boldsymbol{\gamma}}_{1j} - \tau\widehat{\mathbf{R}}_{1j}\boldsymbol{\gamma}_j)^\top(\widehat{\mathbf{R}}_{1j})^{-1}(\widehat{\boldsymbol{\gamma}}_{1j} - \tau\widehat{\mathbf{R}}_{1j}\boldsymbol{\gamma}_j)] \\
&= \mathbb{E}_q[\widehat{\boldsymbol{\gamma}}_{1j}^\top(\widehat{\mathbf{R}}_{1j})^{-1}\widehat{\boldsymbol{\gamma}}_j - 2\tau\boldsymbol{\gamma}_j^\top\widehat{\boldsymbol{\gamma}}_{1j} + \tau^2\boldsymbol{\gamma}_j^\top\widehat{\mathbf{R}}_{1j}\boldsymbol{\gamma}_j] \\
&= \widehat{\boldsymbol{\gamma}}_{1j}^\top(\widehat{\mathbf{R}}_{1j})^{-1}\widehat{\boldsymbol{\gamma}}_{1j} - 2\tau\mathbf{u}_j^\top\widehat{\boldsymbol{\gamma}}_{1j} + \tau^2\mathbf{u}_j^\top\widehat{\mathbf{R}}_{1j}\mathbf{u}_j \\
&\quad + \tau^2(\mathbf{v}_j \odot \mathbf{v}_j)^\top\text{diag}(\widehat{\mathbf{R}}_{1j}) \\
&= (\widehat{\boldsymbol{\gamma}}_{1j} - \tau\widehat{\mathbf{R}}_{1j}\mathbf{u}_j)^\top(\widehat{\mathbf{R}}_{1j})^{-1}(\widehat{\boldsymbol{\gamma}}_{1j} - \tau\widehat{\mathbf{R}}_{1j}\mathbf{u}_j) \\
&\quad + \tau^2(\mathbf{v}_j \odot \mathbf{v}_j)^\top\text{diag}(\widehat{\mathbf{R}}_{1j}),
\end{aligned} \tag{S19}$$

and

$$\begin{aligned}
&\mathbb{E}_q[(\widehat{\boldsymbol{\gamma}}_{2j} - \alpha_j c_j\widehat{\mathbf{R}}_{2j}\boldsymbol{\gamma}_j)^\top(\widehat{\mathbf{R}}_{2j})^{-1}(\widehat{\boldsymbol{\gamma}}_{2j} - \alpha_j c_j\widehat{\mathbf{R}}_{2j}\boldsymbol{\gamma}_j)] \\
&= \mathbb{E}_q[\widehat{\boldsymbol{\gamma}}_{2j}^\top(\widehat{\mathbf{R}}_{2j})^{-1}\widehat{\boldsymbol{\gamma}}_{2j} - 2\alpha_j c_j\boldsymbol{\gamma}_j^\top\widehat{\boldsymbol{\gamma}}_{2j} + \alpha_j^2 c_j^2\boldsymbol{\gamma}_j^\top\widehat{\mathbf{R}}_{2j}\boldsymbol{\gamma}_j] \\
&= \widehat{\boldsymbol{\gamma}}_{2j}^\top(\widehat{\mathbf{R}}_{2j})^{-1}\widehat{\boldsymbol{\gamma}}_{2j} - 2\alpha_j c_j\mathbf{u}_j^\top\widehat{\boldsymbol{\gamma}}_{2j} \\
&\quad + \alpha_j^2 c_j^2\mathbf{u}_j^\top\widehat{\mathbf{R}}_{2j}\mathbf{u}_j + \alpha_j^2 c_j^2(\mathbf{v}_j \odot \mathbf{v}_j)^\top\text{diag}(\widehat{\mathbf{R}}_{2j}) \\
&= (\widehat{\boldsymbol{\gamma}}_{2j} - \alpha_j c_j\widehat{\mathbf{R}}_{2j}\mathbf{u}_j)^\top(\widehat{\mathbf{R}}_{2j})^{-1}(\widehat{\boldsymbol{\gamma}}_{2j} - \alpha_j c_j\widehat{\mathbf{R}}_{2j}\mathbf{u}_j) \\
&\quad + \alpha_j^2 c_j^2(\mathbf{v}_j \odot \mathbf{v}_j)^\top\text{diag}(\widehat{\mathbf{R}}_{2j}).
\end{aligned} \tag{S20}$$

Plugging in these expectations into the first term of ELBO, we obtain

$$
\begin{aligned}
&\mathbb{E}_q[\log \Pr(\widehat{\gamma}_{1j}, \widehat{\gamma}_{2j}, \boldsymbol{\gamma}_j \mid \widehat{\mathbf{R}}_{1j}, \widehat{\mathbf{R}}_{2j}; \boldsymbol{\theta})] \\
&= -\frac{1}{2}\log|\widehat{\mathbf{R}}_{1j}| - \frac{1}{2}(\widehat{\gamma}_{1j} - \tau\widehat{\mathbf{R}}_{1j}\mathbf{u}_j)^\top(\widehat{\mathbf{R}}_{1j})^{-1}(\widehat{\gamma}_{1j} - \tau\widehat{\mathbf{R}}_{1j}\mathbf{u}_j) \\
&\quad - \frac{\tau^2}{2}(\mathbf{v}_j \odot \mathbf{v}_j)^\top \mathrm{diag}(\widehat{\mathbf{R}}_{1j}) \\
&\quad - \frac{1}{2}\log|\widehat{\mathbf{R}}_{2j}| - \frac{1}{2}(\widehat{\gamma}_{2j} - \alpha_j c_j\widehat{\mathbf{R}}_{2j}\mathbf{u}_j)^\top(\widehat{\mathbf{R}}_{2j})^{-1}(\widehat{\gamma}_{2j} - \alpha_j c_j\widehat{\mathbf{R}}_{2j}\mathbf{u}_j) \\
&\quad - \frac{\alpha_j^2 c_j^2}{2}(\mathbf{v}_j \odot \mathbf{v}_j)^\top \mathrm{diag}(\widehat{\mathbf{R}}_{2j}) \\
&\quad - \frac{m_j}{2}\log(2\pi\sigma_{\gamma_j}^2) - \frac{||\mathbf{u}_j||^2 + ||\mathbf{v}_j||^2}{2\sigma_{\gamma_j}^2}.
\end{aligned} \tag{S21}
$$

The second term of ELBO is

$$
\mathbb{E}_q[\log q(\boldsymbol{\gamma}_{jk})] = -\frac{1}{2}\log|2\pi v_{jk}^2| - \frac{1}{2} \tag{S22}
$$

and the complete analytical form of the ELBO is

$$
\begin{aligned}
\mathcal{L}(q) = &-\frac{1}{2}\log|\widehat{\mathbf{R}}_{1j}| - \frac{\tau^2}{2}(\mathbf{v}_j \odot \mathbf{v}_j)^\top \mathrm{diag}(\widehat{\mathbf{R}}_{1j}) \\
&- \frac{1}{2}(\widehat{\gamma}_{1j} - \tau\widehat{\mathbf{R}}_{1j}\mathbf{u}_j)^\top(\widehat{\mathbf{R}}_{1j})^{-1}(\widehat{\gamma}_{1j} - \tau\widehat{\mathbf{R}}_{1j}\mathbf{u}_j) \\
&- \frac{1}{2}\log|\widehat{\mathbf{R}}_{2j}| - \frac{\alpha_j^2 c_j^2}{2}(\mathbf{v}_j \odot \mathbf{v}_j)^\top \mathrm{diag}(\widehat{\mathbf{R}}_{2j}) \\
&- \frac{1}{2}(\widehat{\gamma}_{2j} - \alpha_j c_j\widehat{\mathbf{R}}_{2j}\mathbf{u}_j)^\top(\widehat{\mathbf{R}}_{2j})^{-1}(\widehat{\gamma}_{2j} - \alpha_j c_j\widehat{\mathbf{R}}_{2j}\mathbf{u}_j) \\
&- \frac{m_j}{2}\log(2\pi\sigma_{\gamma_j}^2) - \frac{||\mathbf{u}_j||^2 + ||\mathbf{v}_j||^2}{2\sigma_{\gamma_j}^2} + \frac{1}{2}\sum_{k=1}^{m_j}\log|2\pi v_{jk}^2| + \frac{m_j}{2}.
\end{aligned} \tag{S23}
$$

Grouping the constant terms together gives the following simplified form of the ELBO

$$
\begin{aligned}
\mathcal{L}(q) = \tau\mathbf{u}_j^\top\widehat{\gamma}_{1j} &- \frac{1}{2}\tau^2\mathbf{u}_j^\top\widehat{\mathbf{R}}_{1j}\mathbf{u}_j - \frac{\tau^2}{2}(\mathbf{v}_j \odot \mathbf{v}_j)^\top \mathrm{diag}(\widehat{\mathbf{R}}_{1j}) \\
&+ \alpha_j c_j\mathbf{u}_j^\top\widehat{\gamma}_{2j} - \frac{1}{2}\alpha_j^2 c_j^2\mathbf{u}_j^\top\widehat{\mathbf{R}}_{2j}\mathbf{u}_j - \frac{\alpha_j^2 c_j^2}{2}(\mathbf{v}_j \odot \mathbf{v}_j)^\top \mathrm{diag}(\widehat{\mathbf{R}}_{2j}) \\
&- \frac{m_j}{2}\log(2\pi\sigma_{\gamma_j}^2) - \frac{||\mathbf{u}_j||^2 + ||\mathbf{v}_j||^2}{2\sigma_{\gamma_j}^2} + \frac{1}{2}\sum_{k=1}^{m_j}\log|2\pi v_{jk}^2| + \text{constant}.
\end{aligned} \tag{S24}
$$

1.4.3 Estimation of model parameters

The parameter updates are obtained by setting their corresponding derivatives equal to zero:

$$\begin{aligned}
\frac{\partial \mathcal{L}(q)}{\partial \sigma^2_{\gamma_j}} &= -\frac{m_j}{2\sigma^2_{\gamma_j}} + \frac{||\mathbf{u}_j||^2 + ||\mathbf{v}_j||^2}{2\sigma^4_{\gamma_j}} = 0, \\
\frac{\partial \mathcal{L}(q)}{\partial \tau} &= \mathbf{u}_j^\top \widehat{\boldsymbol{\gamma}}_{1j} - \tau \mathbf{u}_j^\top \widehat{\mathbf{R}}_{1j} \mathbf{u}_j - \tau (\mathbf{v}_j \odot \mathbf{v}_j)^\top \text{diag}(\widehat{\mathbf{R}}_{1j}) = 0, \\
\frac{\partial \mathcal{L}(q)}{\partial \alpha_j} &= c_j \mathbf{u}_j^\top \widehat{\boldsymbol{\gamma}}_{2j} - \alpha_j c_j^2 \mathbf{u}_j^\top \widehat{\mathbf{R}}_{2j} \mathbf{u}_j - \alpha_j c_j^2 (\mathbf{v}_j \odot \mathbf{v}_j)^\top \text{diag}(\widehat{\mathbf{R}}_{2j}) = 0.
\end{aligned} \tag{S25}$$

Hence, the parameter updates are

$$\begin{aligned}
\sigma^2_{\gamma_j} &= \frac{||\mathbf{u}_j||^2 + ||\mathbf{v}_j||^2}{m_j}, \\
\alpha_j &= \frac{\mathbf{u}_j^\top \widehat{\boldsymbol{\gamma}}_{2j}}{c_j \mathbf{u}_j^\top \widehat{\mathbf{R}}_{2j} \mathbf{u}_j + c_j (\mathbf{v}_j \odot \mathbf{v}_j)^\top \text{diag}(\widehat{\mathbf{R}}_{2j})}, \\
\tau &= \frac{\mathbf{u}_j^\top \widehat{\boldsymbol{\gamma}}_{1j}}{\mathbf{u}_j^\top \widehat{\mathbf{R}}_{1j} \mathbf{u}_j + (\mathbf{v}_j \odot \mathbf{v}_j)^\top \text{diag}(\widehat{\mathbf{R}}_{1j})}.
\end{aligned} \tag{S26}$$

In practice, we may not be able to observe the data-dependent value c_j. In this case, we treat $c_j \alpha_j$ as a single parameter and update its value accordingly. The subsequent likelihood ratio test is unchanged as testing $\alpha_j = 0$ is equivalent to testing $c_j \alpha_j = 0$ since c_j (a function of the GWAS trait sample variance, GWAS sample size, eQTL gene expression sample variance and eQTL sample size) is not equal to 0.

1.4.4 Reduction to original model

To obtain the original model from the expanded model, we perform the reduction steps

$$\begin{aligned}
\alpha_j &= \frac{\alpha_j}{\tau}, \\
\sigma^2_{\gamma_j} &= \tau^2 \sigma^2_{\gamma_j}, \\
u_{jk} &= \tau u_{jk}, \\
v_{jk} &= \tau^2 v_{jk},
\end{aligned} \tag{S27}$$

and set $\tau = 1$.

The variational PX-VBEM algorithm for CoMM-S^4 can be summarized as

Algorithm 1: The PX-VBEM algorithm for CoMM-S^4

Initialization: $\alpha_j = 0, \tau = 1.$
repeat
    E-step: Set $\alpha_j = \alpha_j^{(t)}$ and $\sigma^2_{\gamma_j} = (\sigma^2_{\gamma_j})^{(t)}$, and update the parameters of variational distribution γ_j using the formulas in (S15).
    M-step: Set $\frac{\partial \mathcal{L}(q)}{\partial \boldsymbol{\theta}} = 0$, and update the parameters $\sigma^2_{\gamma_j}$, α_j and τ using the formulas in (S26).
    Reduction-step: $\alpha_j^{(t+1)} = \frac{\alpha_j^{(t+1)}}{\tau^{(t+1)}}$, $(\sigma_{\gamma_j}^{(t+1)})^2 = (\tau^{(t+1)})^2(\sigma_{\gamma_j}^{(t+1)})^2$
until *Convergence*;

1.5 Linkage disequilibrium estimation

The log-likelihood is a function of the SNP correlation matrices $\widehat{\mathbf{R}}_{1j}$ and $\widehat{\mathbf{R}}_{2j}$ that correspond to the eQTL and GWAS datasets, respectively. We briefly describe the estimation procedure for $\widehat{\mathbf{R}}_{1j}$. The procedure is the same for $\widehat{\mathbf{R}}_{2j}$. We first calculate the empirical correlation matrix $\widehat{\mathbf{R}}_{1j}^{\text{emp}} = [r_{ik}] \in \mathbb{R}^{m_j \times m_j}$ with $r_{ik} = \frac{\mathbf{w}_{1ji}^T \mathbf{w}_{1jk}}{\sqrt{(\mathbf{w}_{1ji}^T \mathbf{w}_{1ji})(\mathbf{w}_{1jk}^T \mathbf{w}_{1jk})}}$, where $\mathbf{w}_{1jk}$ is the genotype vector of eQTL for the k-th genetic variant within the j-th gene. To make the estimated correlation matrix positive definite, a simple shrinkage estimator (Schäfer and Strimmer, 2005) is applied to obtain $\widehat{\mathbf{R}}_{1j}$ as $\widehat{\mathbf{R}}_{1j} = \lambda \widehat{\mathbf{R}}_{1j}^{\text{emp}} + (1-\lambda)\mathbf{I}_{m_j}$, where $\lambda \in [0,1]$ is the shrinkage intensity. We have tested with different $\lambda \in [0.8, 0.95]$ and the results are robust to the choice of λ.

2 STATISTICAL INFERENCE

2.1 Likelihood ratio test to evaluate expression-trait association

We perform a likelihood ratio test for expression-trait association:

$$\mathcal{H}_0 : \alpha_j = 0 \qquad \mathcal{H}_a : \alpha_j \neq 0. \tag{S28}$$

This is equivalent to testing

$$\mathcal{H}_0 : c_j\alpha_j = 0 \qquad \mathcal{H}_a : c_j\alpha_j \neq 0, \tag{S29}$$

since $c_j \neq 0$. The test statistic for the j-th gene is

$$\Lambda_j = 2\big(\log \Pr(\widehat{\gamma}_{1j}, \widehat{\gamma}_{2j} \mid \widehat{\mathbf{R}}_{1j}, \widehat{\mathbf{R}}_{2j}; \hat{\boldsymbol{\theta}}^{\text{ML}}) - \log \Pr(\widehat{\gamma}_{1j}, \widehat{\gamma}_{2j} \mid \widehat{\mathbf{R}}_{1j}, \widehat{\mathbf{R}}_{2j}; \hat{\boldsymbol{\theta}}_0^{\text{ML}})\big), \tag{S30}$$

where $\hat{\boldsymbol{\theta}}_0^{\text{ML}}$ and $\hat{\boldsymbol{\theta}}^{\text{ML}}$ are vectors of parameter estimated obtained by maximizing the marginal likelihood under the null hypothesis $\mathcal{H}_0$ and alternative hypothesis $\mathcal{H}_a$. The test statistic asymptotically follows the $\chi^2_{\text{df}=1}$ under the null hypothesis (Van der Vaart, 2000).

2.2 Evaluation of log-likelihood

The marginal log-likelihood is

$$\begin{aligned}&\log \Pr(\widehat{\gamma}_{1j}, \widehat{\gamma}_{2j} \mid \widehat{\mathbf{R}}_{1j}, \widehat{\mathbf{R}}_{2j}; \boldsymbol{\theta}) \\ &= \mathbb{E}_p[\log \Pr(\widehat{\gamma}_{1j}, \widehat{\gamma}_{2j}, \boldsymbol{\gamma}_j \mid \widehat{\mathbf{R}}_{1j}, \widehat{\mathbf{R}}_{2j}; \boldsymbol{\theta})] - \mathbb{E}_p[\log p(\boldsymbol{\gamma}_j \mid \widehat{\gamma}_{1j}, \widehat{\gamma}_{2j}, \widehat{\mathbf{R}}_{1j}, \widehat{\mathbf{R}}_{2j}; \boldsymbol{\theta})].\end{aligned} \tag{S31}$$

where the expectation is taken over the posterior distribution of the latent variable $\boldsymbol{\gamma}_j$. The posterior distribution is proportional to the complete-data log-likelihood,

$$\begin{aligned}p(\boldsymbol{\gamma}_j \mid \widehat{\gamma}_{1j}, \widehat{\gamma}_{2j}, \widehat{\mathbf{R}}_{1j}, \widehat{\mathbf{R}}_{2j}, \boldsymbol{\theta}) &\propto p(\widehat{\gamma}_{1j}, \widehat{\gamma}_{2j} \mid \boldsymbol{\gamma}_j, \widehat{\mathbf{R}}_{1j}, \widehat{\mathbf{R}}_{2j}, \boldsymbol{\theta}) f(\boldsymbol{\gamma}_j \mid \boldsymbol{\theta}) \\ &\propto \exp\left[-\frac{1}{2}(\widehat{\gamma}_{1j} - \widehat{\mathbf{R}}_{1j}\boldsymbol{\gamma}_j)^\top \widehat{\mathbf{R}}_{1j}^{-1}(\widehat{\gamma}_{1j} - \widehat{\mathbf{R}}_{1j}\boldsymbol{\gamma}_j)\right] \\ &\quad \exp\left[-\frac{1}{2}(\widehat{\gamma}_{2j} - \alpha_j c_j \widehat{\mathbf{R}}_{2j}\boldsymbol{\gamma}_j)^\top \widehat{\mathbf{R}}_{2j}^{-1}(\widehat{\gamma}_{2j} - \alpha_j c_j \widehat{\mathbf{R}}_{2j}\boldsymbol{\gamma}_j)\right] \\ &\quad \exp\left[-\frac{1}{2}\boldsymbol{\gamma}_j^\top \left(\sigma^2_{\gamma_j}\mathbf{I}_{m_j}\right)^{-1} \boldsymbol{\gamma}_j\right],\end{aligned} \tag{S32}$$

and has the form of a normal distribution. Hence, the posterior distribution is $\boldsymbol{\gamma}_j \sim \mathcal{N}(\boldsymbol{\mu}_j, \boldsymbol{\Sigma}_j)$, where

$$\begin{aligned}\boldsymbol{\Sigma}_j &= \left(\widehat{\mathbf{R}}_{1j} + \alpha_j^2 c_j^2 \widehat{\mathbf{R}}_{2j} + \frac{1}{\sigma^2_{\gamma_j}}\mathbf{I}_{m_j}\right)^{-1} \\ \boldsymbol{\mu}_j &= \boldsymbol{\Sigma}_j\left(\widehat{\gamma}_{1j} + \alpha_j c_j \widehat{\gamma}_{2j}\right),\end{aligned} \tag{S33}$$

and the marginal log-likelihood is

$$\begin{aligned}\widetilde{\mathcal{L}}(\boldsymbol{\theta}) &= \boldsymbol{\mu}_j^T \widehat{\boldsymbol{\gamma}}_{1j} - \frac{1}{2}\boldsymbol{\mu}_j^T \widehat{\mathbf{R}}_{1j}\boldsymbol{\mu}_j - \frac{1}{2}\text{tr}(\boldsymbol{\Sigma}_j \widehat{\mathbf{R}}_{1j}) \\ &+ \alpha_j c_j \boldsymbol{\mu}_j^T \widehat{\boldsymbol{\gamma}}_{2j} - \frac{1}{2}\alpha_j^2 c_j^2 \boldsymbol{\mu}_j^T \widehat{\mathbf{R}}_{2j}\boldsymbol{\mu}_j - \frac{\alpha_j^2 c_j^2}{2}\text{tr}(\boldsymbol{\Sigma}_j \widehat{\mathbf{R}}_{2j}) \\ &- \frac{m_j}{2}\log(2\pi\sigma_{\gamma_j}^2) - \frac{||\boldsymbol{\mu}_j||^2 + \text{tr}(\boldsymbol{\Sigma}_j)}{2\sigma_{\gamma_j}^2} + \frac{1}{2}\log|\boldsymbol{\Sigma}_j| + \text{constant.}\end{aligned} \tag{S34}$$

3 SIMULATION

3.1 General simulation settings and notation

We let n_1, n_2, n_3 and n_4 denote the sample sizes of eQTL dataset, GWAS dataset, eQTL LD reference panel dataset, and GWAS LD reference panel dataset, respectively. The genotype data is generated using parameters that control the SNP correlation matrix, and the minor allele frequencies. We first generate a raw data matrix using a multivariate normal distribution $\mathcal{N}(\mathbf{0}, \Sigma(\rho))$, where $\Sigma(\rho)$ is an auto-regressive correlation structure, and the parameter ρ determines the strength of correlations among the SNPs ($\rho \in \{0.2, 0.5, 0.8\}$). Next, we use the uniform distribution $\mathcal{U}(0.05, 0.5)$ to generate the minor allele frequencies (MAF). Based on the MAF and assuming Hardy-Weinberg equilibrium, the continuous values in the raw data matrix is discretized into trinary values $\{0, 1, 2\}$. All four genotype matrices, $\mathbf{W}_{1j}$, $\mathbf{W}_{2j}$, $\mathbf{W}_{1j}^{\mathrm{ref}}$ and $\mathbf{W}_{2j}^{\mathrm{ref}}$ (where $\mathbf{W}_{1j}$, $\mathbf{W}_{2j}$ are as defined in Section 1.2, and $\mathbf{W}_{1j}^{\mathrm{ref}}$ and $\mathbf{W}_{2j}^{\mathrm{ref}}$ are genotype data for the eQTL and GWAS LD reference panels) are generated in this manner.

We generate the gene expression according to $\mathbf{y}_j = \mathbf{W}_{1j}\boldsymbol{\gamma}_j + \mathbf{e}_1$, where $\mathbf{e}_1 \sim \mathcal{N}(\mathbf{0}, \sigma_{e_1}^2 \mathbf{I}_{n_1})$ and the effect size $\boldsymbol{\gamma}_j$ is generated from a spike-slab distribution $\gamma_{jk} \sim \pi\mathcal{N}(\mathbf{0}, \sigma_{\gamma_j}^2) + (1-\pi)\delta_0$, π is the sparsity level, δ_0 denotes a Dirac delta mass function at 0, and k is the index for genetic variants within gene j. To simulate distinct scenarios, we consider equally-spaced cellular heritability levels ($h_C^2 \in \{0.01, 0.03, 0.05, 0.07, 0.09\}$), which reflect the proportion of gene expression variability accounted for by SNP variability. We also consider various sparsity levels (0.1, 0.2, 0.3, 0.4, 0.5 and 1), which reflect the proportion of SNPs with SNP-expression effects. We generate the complex traits according to $\mathbf{z} = \alpha_j \mathbf{W}_{2j}\boldsymbol{\gamma}_j + \mathbf{e}_2$ and the number of cis-SNPs (m_j) is set to be 100. The trait-level heritability (h_T^2) is set to be 0 under the null hypothesis and 0.001, 0.002, 0.003 under the alternative hypothesis. The corresponding summary statistics were generated by applying a simple linear regression to the individual-level eQTL and GWAS dataset, respectively.

3.2 Comparison between CoMM-S^4 and CoMM-S^2

Here, we compare the LRT test statistics from CoMM-S^4 and CoMM-S^2 to demonstrate the performance of CoMM-S^4. The major difference lies in that CoMM-S^4 harnesses the summary-level eQTL summary data rather than the individual-level eQTL data. In this simulation study, we consider the model setting n_1 = 5,000, n_2 = 5,000, n_3 = 400, n_4 = 400, m_j=100, $\rho \in \{0.2, 0.5, 0.8\}$, $h_C^2 \in \{0.01, 0.03, 0.05, 0.07, 0.09\}$ and $h_T^2 \in \{0, 0.001, 0.002, 0.003\}$. In these simulations, the reference panel for the eQTL data is subsampled from the individual-level eQTL dataset, and the reference panel for the GWAS data is subsampled from the individual-level GWAS dataset.

The simulation results indicate that the LRT test statistics from CoMM-S^4 and CoMM-S^2 analyses are similar. We regress the CoMM-S^4 test statistic against the CoMM-S^2 statistic, using simple linear regression. The slope ranges from 0.88 to 1 and R^2 ranges from 0.80 to 1 (Figures S1-S6).

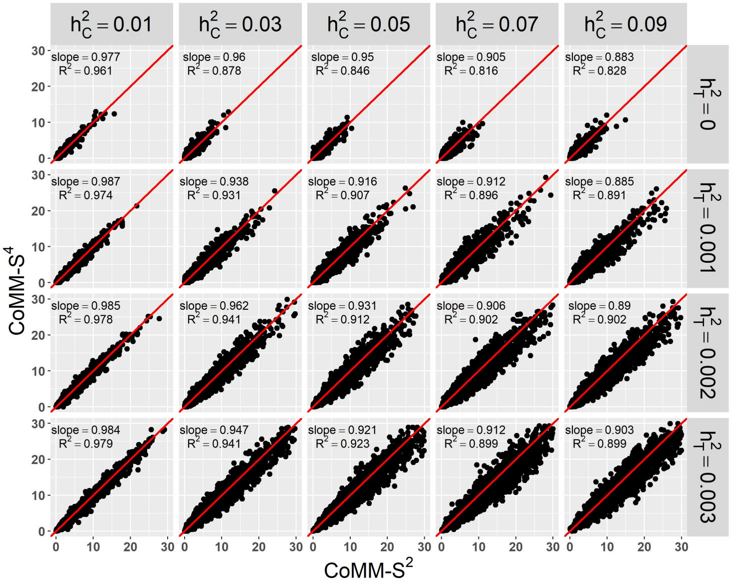

Figure S1: The scatterplot of CoMM-S^4 vs CoMM-S^2. The simulation settings are n_1 = 5,000, n_2 = 5,000, n_3 = 400, n_4 = 400, $m_j = 100$, $\rho = 0.2$, $\pi = 0.2$, and 2000 replicates were used.

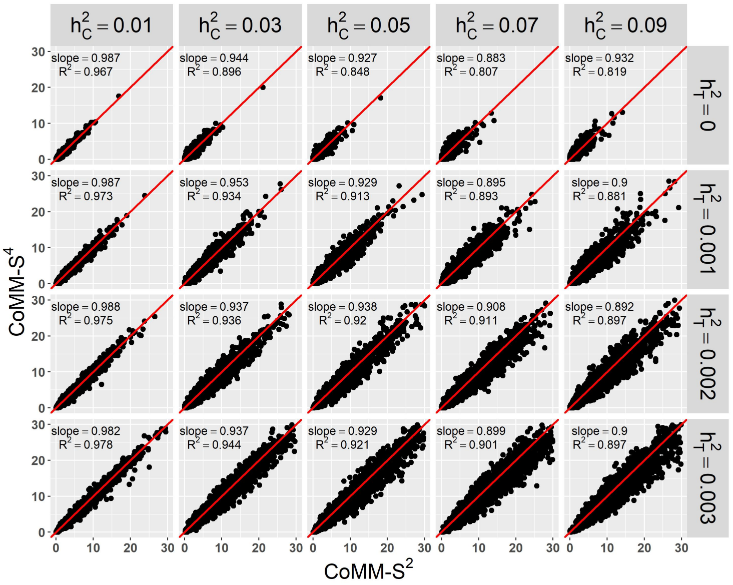

Figure S2: The scatterplot of CoMM-S^4 vs CoMM-S^2. The simulation settings are n_1 = 5,000, n_2 = 5,000, n_3 = 400, n_4 = 400, $m_j = 100$, $\rho = 0.2$, $\pi = 1$, and 2000 replicates were used.

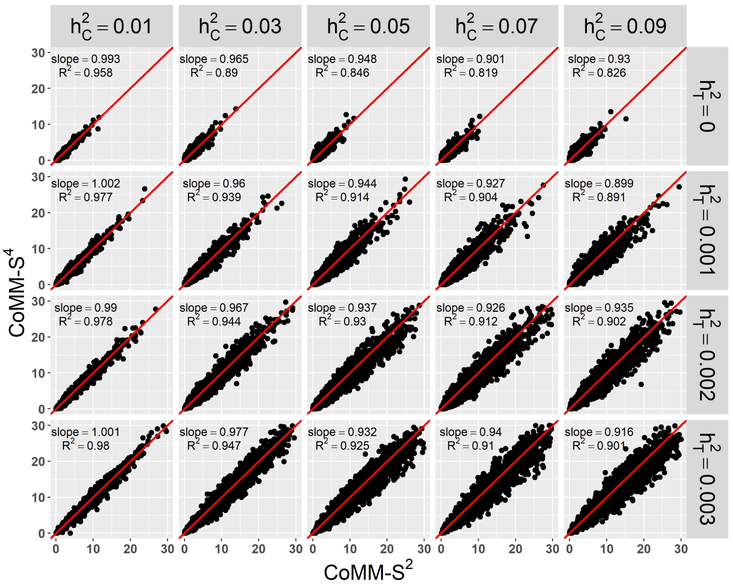

Figure S3: The scatterplot of CoMM-S^4 vs CoMM-S^2. The simulation settings are n_1 = 5,000, n_2 = 5,000, n_3 = 400, n_4 = 400, $m_j = 100$, $\rho = 0.5$, $\pi = 0.2$, and 2000 replicates were used.

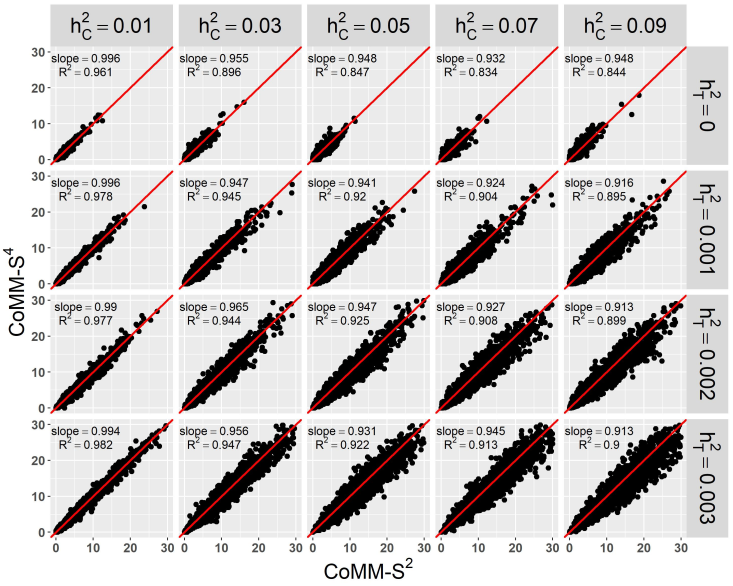

Figure S4: The scatterplot of CoMM-S^4 vs CoMM-S^2. The simulation settings are n_1 = 5,000, n_2 = 5,000, n_3 = 400, n_4 = 400, $m_j = 100$, $\rho = 0.5$, $\pi = 1$, and 2000 replicates were used.

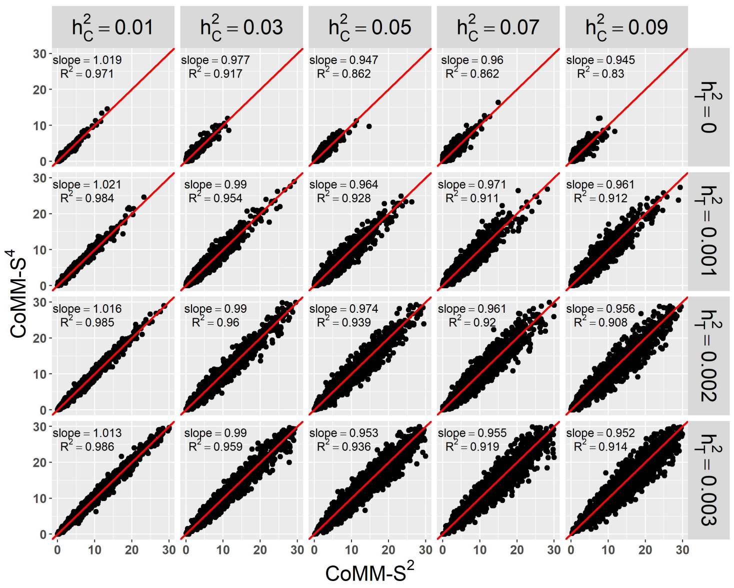

Figure S5: The scatterplot of CoMM-S^4 vs CoMM-S^2. The simulation settings are n_1 = 5,000, n_2 = 5,000, n_3 = 400, n_4 = 400, $m_j = 100$, $\rho = 0.8$, $\pi = 0.2$, and 2000 replicates were used.

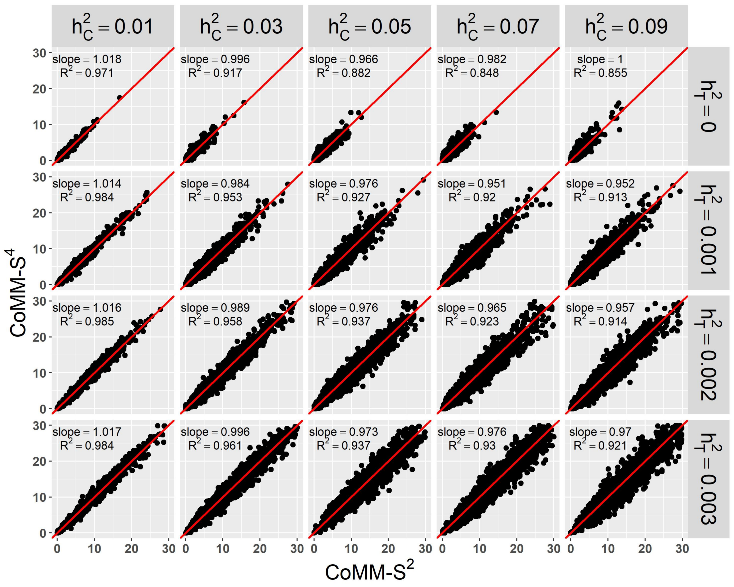

Figure S6: The scatterplot of CoMM-S^4 vs CoMM-S^2. The simulation settings are n_1 = 5,000, n_2 = 5,000, n_3 = 400, n_4 = 400, $m_j = 100$, $\rho = 0.8$, $\pi = 1$, and 2000 replicates were used.

3.3 Type-I error of CoMM-S^4

We evaluate the type-I error of CoMM-S^4 by inspecting the QQ plots, with data generated under $\alpha_j = 0$ and $h_T^2 = 0$. For all simulations, we set n_1 = 5,000, n_2 = 5,000, n_3 = 400, n_4 = 400, $\rho \in \{0.2, 0.5, 0.8\}$, $h_C^2 \in \{0.01, 0.03, 0.05, 0.07, 0.09\}$ and signal sparsity at either 0.2 or 1. In these simulations, the LD reference panel for eQTL is subsampled from the individual-level eQTL dataset, and the LD reference panel for GWAS is subsampled from the individual-level GWAS dataset.

The QQ plots indicate that the p-values behave as expected under the null hypothesis, for all SNP correlation parameters considered ($\rho \in \{0.2, 0.5, 0.8\}$, Figures S7-S9).

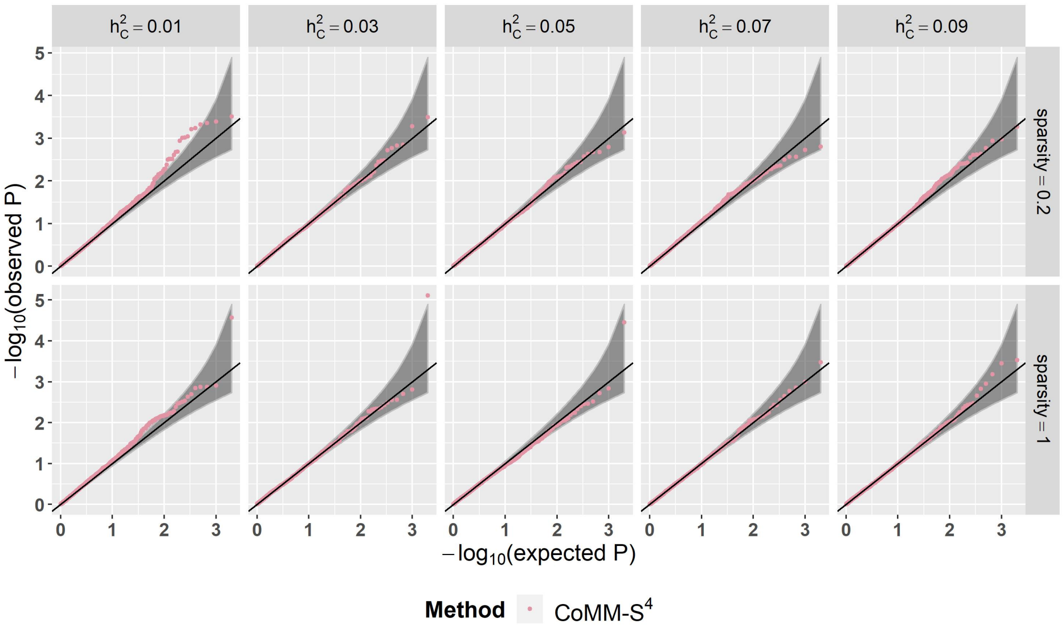

Figure S7: The QQ plot of CoMM-S^4. The simulation settings are n_1 = 5,000, n_2 = 5,000, n_3 = 400, n_4 = 400, $\rho = 0.2$, and 2000 replicates were used.

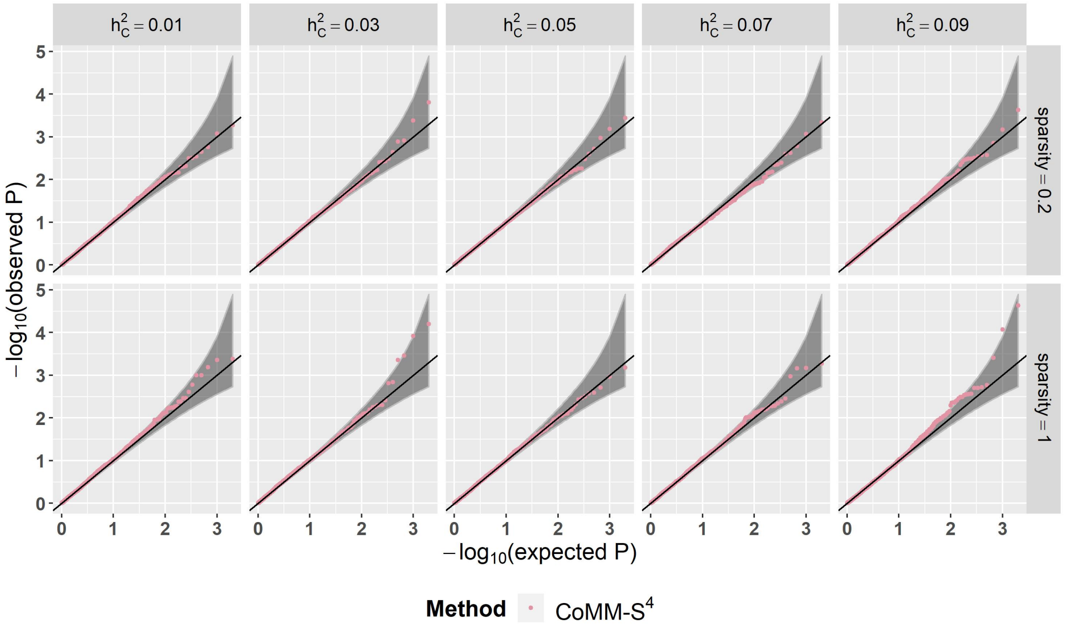

Figure S8: The QQ plot of CoMM-S^4. The simulation settings are n_1 = 5,000, n_2 = 5,000, n_3 = 400, n_4 = 400, $\rho = 0.5$, and 2000 replicates were used.

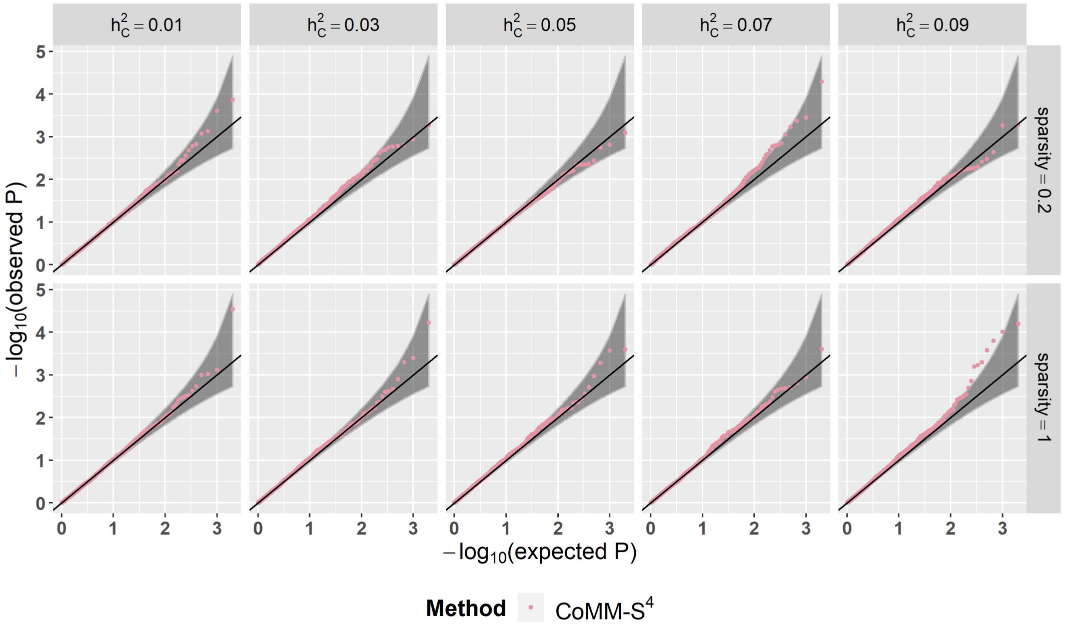

Figure S9: The QQ plot of CoMM-S^4. The simulation settings are n_1 = 5,000, n_2 = 5,000, n_3 = 400, n_4 = 400, $\rho = 0.8$, and 2000 replicates were used.

3.4 Comparison with CoMM-S^2 and S-PrediXcan

We evaluate the power of CoMM-S^4, CoMM-S^2, S-PrediXcan (ridge) and S-PrediXcan (elastic net). For all simulations, we set the number of eQTL samples n_1 = 500, the number of GWAS samples n_2 = 10,000, the number of eQTL LD reference panel samples n_3 = 400, and the number of GWAS LD reference panel samples n_4 = 400. We set the cellular heritability $h_C^2 \in \{0.01, 0.03, 0.05, 0.07, 0.09\}$, the trait heritability $h_T^2 \in \{0, 0.001, 0.002, 0.003\}$, the number of cis-SNPs $m_j = 100$, and signal sparsity in $\{0.1, 0.2, 0.3, 0.4, 0.5, 1\}$. The minor allele frequencies (MAF) are generated from $\mathcal{U}(0.05, 0.50)$. Three simulation scenarios are considered:

1. eQTL and GWAS samples are from the same population. We simulate the MAF and SNP correlations for a single population. The strength of correlation between SNPs is set at $\rho \in \{0.2, 0.5, 0.8\}$. The genotypes of (a) eQTL samples, (b) eQTL LD reference panel samples, (c) GWAS samples and (d) GWAS LD reference panel samples are generated based on the population parameters. The gene expression architecture (causal eQTLs) are the same for both the GWAS and eQTL datasets.
2. eQTL and GWAS populations have distinct LD structures. The MAF of the eQTL population are drawn from $\mathcal{U}_1(0.05, 0.50)$. The SNP correlations are derived from the 1000 Genomes Project European (EUR) samples as follows:
   - Genes with at least m_j SNPs within 1Mb from the center of the gene are randomly sampled from the eQTLGen dataset. For each of these genes, m_j SNPs are randomly selected from its SNP set.
   - The genetic correlations between the m_j SNPs are calculated using EUR samples from the 1000 Genomes Project.

   The MAF and SNP correlation parameters of this first population are used to generate the genotypes of (i) eQTL samples and (ii) eQTL LD reference panel samples. Similarly, the MAF of the GWAS population are drawn from $\mathcal{U}_2(0.05, 0.50)$ and the SNP correlations are sampled from the 1000 Genomes Project Japanese (JPT) samples. The MAF and SNP correlation parameters of this second population are used to generate the genotypes of (i) GWAS samples and (ii) GWAS LD reference panel samples. The simulated causal eQTLs are the same for the GWAS and eQTL datasets.
3. eQTL and GWAS populations have distinct LD structures, and differing gene expression architectures. The genotypes are generated in the same way as in scenario 2. Moreover, differing gene expression architectures in the GWAS and eQTL populations are simulated as follows:
   - For each gene, we first simulated x causal SNPs for the eQTL population.
   - Half of these x SNPs are randomly selected to have SNP-expression effects for the GWAS population.
   - The remaining $0.5x$ causal SNPs for the GWAS population are drawn from the eQTL population's non-causal SNPs.

   The number of SNPs with SNP-expression effect (x) is determined by the sparsity parameter.

3.4.1 eQTL and GWAS samples are from the same population

The empirical type I error ($h_T^2 = 0$) and power ($h_T^2 > 0$) of CoMM-S^4 is comparable to that of CoMM-S^2, S-PrediXcan (ridge) and S-PrediXcan (elastic net), for all SNP correlation parameters considered ($\rho \in \{0.2, 0.5, 0.8\}$, Figures S10-S12). A p-value threshold of 0.05 is used to identify statistically significant genes. The improvement in power of CoMM-S^4 over S-PrediXcan is largest when the cellular heritability, which reflects the strength of the SNP-expression relationship, is small ($h_C^2 = 0.01$) .

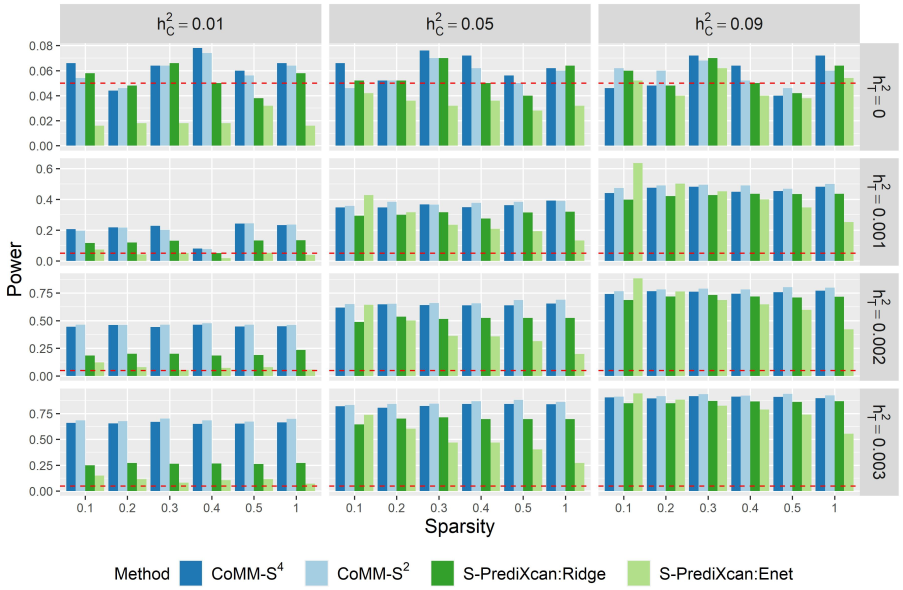

Figure S10: The empirical type I error ($h_T^2 = 0$) and power ($h_T^2 > 0$) of CoMM-S^4, CoMM-S^2, S-PrediXcan (ridge) and S-PrediXcan (elastic net) across 500 replications. The model setting is n_1 = 500, n_2 = 10,000, n_3 = 400, n_4 = 400, $\rho = 0.2$.

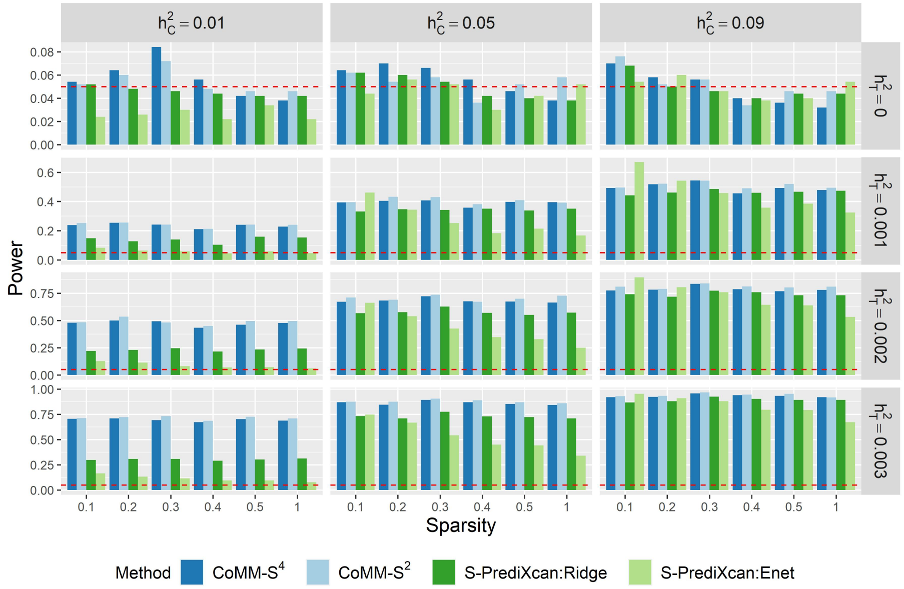

Figure S11: The empirical type I error ($h_T^2 = 0$) and power ($h_T^2 > 0$) of CoMM-S[4], CoMM-S[2], S-PrediXcan (ridge) and S-PrediXcan (elastic net) across 500 replications. The model setting is n_1 = 500, n_2 = 10,000, n_3 = 400, n_4 = 400, $\rho = 0.5$.

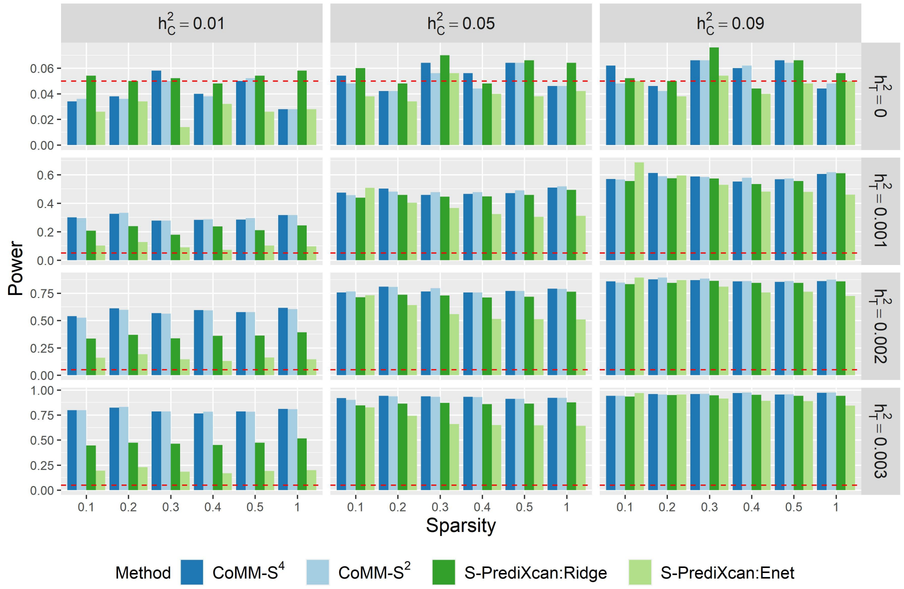

Figure S12: The empirical type I error ($h_T^2 = 0$) and power ($h_T^2 > 0$) of CoMM-S[4], CoMM-S[2], S-PrediXcan (ridge) and S-PrediXcan (elastic net) across 500 replications. The model setting is n_1 = 500, n_2 = 10,000, n_3 = 400, n_4 = 400, $\rho = 0.8$.

3.4.2 eQTL and GWAS populations have distinct LD structures

SNP correlations for the eQTL and GWAS populations were derived from the European samples (EUR) and Japanese samples (JPT), respectively, in the 1000 Genomes Project. The simulations indicate that the empirical type I error ($h_T^2 = 0$) and power ($h_T^2 > 0$) of CoMM-S^4 of CoMM-S^4 is comparable to that of CoMM-S^2, S-PrediXcan (ridge) and S-PrediXcan (elastic net).

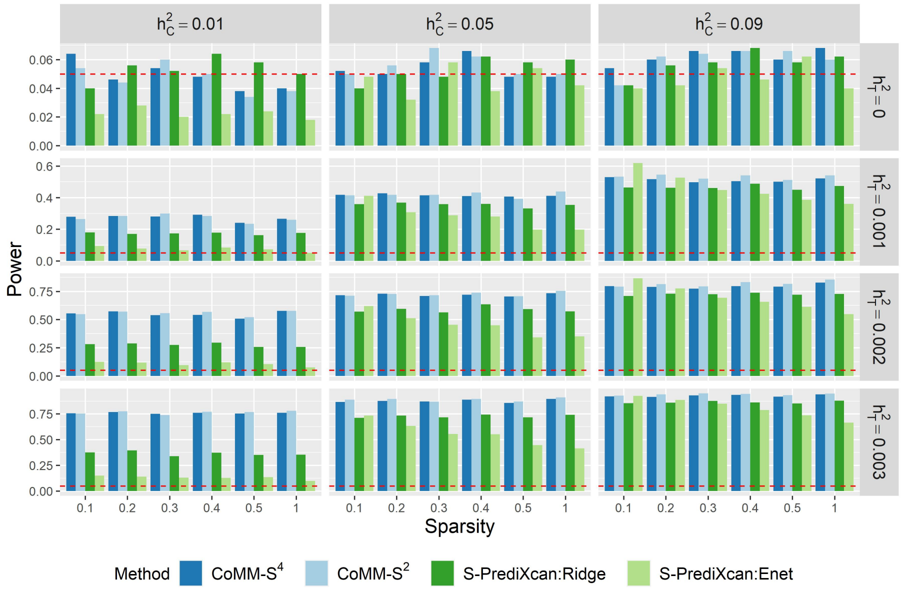

Figure S13: The empirical type I error ($h_T^2 = 0$) and power ($h_T^2 > 0$) of CoMM-S^4, CoMM-S^2, S-PrediXcan (ridge) and S-PrediXcan (elastic net) across 500 replications. The model setting is n_1 = 500, n_2 = 10,000, n_3 = 400, n_4 = 400. Sparsity refers to the percentage of effect SNPs in the eQTL analyses. The two populations have different LD structures.

3.4.3 eQTL and GWAS populations have distinct LD structures, and different gene expression architectures

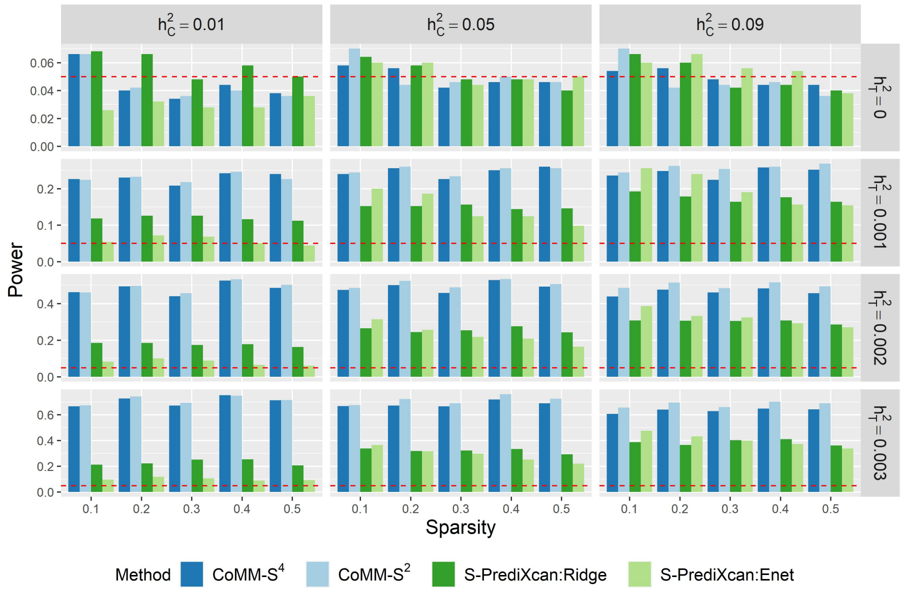

Figure S14: The empirical type I error ($h_T^2 = 0$) and power ($h_T^2 > 0$) of CoMM-S^4, CoMM-S^2, S-PrediXcan (ridge) and S-PrediXcan (elastic net) across 500 replications. The model setting is n_1 = 500, n_2 = 10,000, n_3 = 400, n_4 = 400. Sparsity refers to the percentage of effect SNPs in the eQTL analyses. To simulate different genetic architectures, only 50% of the effect SNPs (in the SNP-expression relationship) are shared between the two populations. The two populations have different LD structures.

4 REAL DATA ANALYSIS

4.1 NFBC1966

In the real data analysis, we apply CoMM-S^4 to the NFBC1966 dataset. The summary-level NFBC1966 data is generated by applying the simple linear regression to individual-level NFBC1966 using software plink. The eQTL summary statistics were obtained from eQTLGen Consortium cis-eQTLs. The eQTL LD reference panel consists of European samples from the 1000 Genomes Project, and the GWAS reference panel consists of 400 subsamples from NFBC1966. The QQ plots are illustrated in Figure S15.

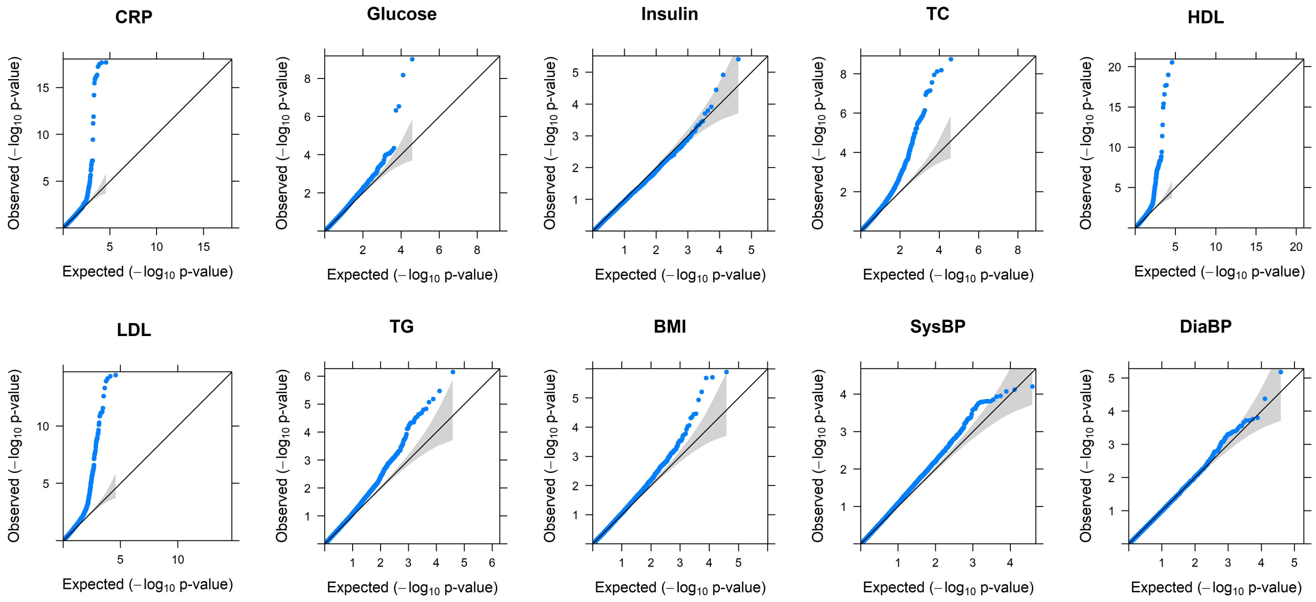

Figure S15: The QQ plot of p-values from CoMM-S^4 analyses for 10 NFBC traits.

4.2 Biobank Japan

We apply CoMM-S^4 to GWAS summary statistics for 9 traits in Biobank Japan (BBJ) (Ishigaki et al., 2020). Whole-blood cis-eQTL summary statistics from two studies, eQTLGen (Võsa et al., 2018) and GTEx (v8) (The GTEx Consortium, 2020), were used to assess the robustness of TWAS results to choice of eQTL dataset. The GTEx and eQTLGen datasets contain association results for 19,599 and 19,176 genes respectively, of which 16,692 genes are in common. Linkage disequilibrium corresponding to the GWAS and eQTL datasets were estimated using Japanese (JPT) and European (EUR) samples from the 1000 Genomes Project (The 1000 Genomes Project Consortium, 2015), respectively. For the TWAS analyses (CoMM-S^4 and later S-PrediXcan), we retain only those SNPs that (i) passed QC in HapMap3 (The International HapMap 3 Consortium, 2010), (ii) had MAF $\geq$ 0.05 in the JPT samples, and (iii) had MAF $\geq$ 0.05 in the EUR samples in the 1000 Genomes Project.

The genomic inflation factors for the CoMM-S^4 analyses, as well as the number of associated genes, are listed in Table S1.

	eQTLGen		GTEx		eQTLGen and GTEx
	GIF	No. associated genes (No. in GTEx)	GIF	No. associated genes (No. in eQTLGen)	No. common associated genes (sig. in BBJ GWAS; reported in GWAS Catalog)
Graves' disease	1.17	283 (247)	1.09	454 (364)	245 (125; 7)
Rheumatoid arthritis	1.30	266 (230)	1.26	402 (323)	220 (134; 22)
Chronic hepatitis B	1.06	148 (133)	0.87	211 (172)	132 (70; 6)
Chronic hepatitis C	1.09	73 (66)	1.00	163 (145)	64 (4; 1)
Ischemic stroke	1.25	23 (21)	1.24	60 (56)	19 (3; 3)
Congestive heart failure	1.18	4 (2)	1.13	10 (9)	1 (0; 0)
Peripheral artery disease	1.13	13 (10)	0.99	45 (37)	7 (0; 0)
Cerebral aneurysm	1.11	4 (4)	0.99	6 (6)	2 (0; 0)
Osteoporosis	1.07	2 (2)	0.93	7 (6)	1 (0; 0)

Table S1. The genomic inflation factor and number of associated genes (p-value $< 5 \times 10^{-6}$) for 9 traits in the Biobank Japan dataset. Two eQTL datasets were used: eQTLGen and GTEx. In parentheses are the number of associated genes that are also present in the other eQTL dataset's gene set. The last column shows the number of associated genes that are common to both the eQTLGen and GTEx analyses; in parentheses are the number of associated genes that are statistically significant in the GWAS analysis (p-value $< 5 \times 10^{-8}$), and the number of associated genes reported in the GWAS Catalog.

In addition, we compare the CoMM-S^4 results with S-PrediXcan (elastic net) results for the 9 Biobank Japan traits. For S-PrediXcan, gene expression prediction weights for GTEx (v8) whole blood were obtained from the elastic net model in PredictDB (http://predictdb.org/). The covariance matrix supplied to S-PrediXcan for the calculation of test statistics is based on Japanese samples in the 1000 Genomes Project. To allow for fair comparison, we consider only genes that are common to both the CoMM-S^4 and S-PrediXcan analyses.

Compared with S-PrediXcan, CoMM-S^4 (eQTL data: GTEx (v8) whole blood) identifies a similar number of statistically significant genes for 5 Biobank Japan traits (cerebral aneurysm, congestive heart failure, ischemic stroke, peripheral artery disease, and osteoporosis), and more statistically significant genes for 4 Biobank Japan traits (Graves' disease, rheumatoid arthritis, chronic hepatitis C, and chronic hepatitis B) (Table S2). The tail behaviour in the QQ plots indicate that the p-values tend to be smaller for statistically significant genes (Figure S16). The higher number of identified genes in real traits is consistent with the higher power demonstrated in simulations.

The QQ plots show inflation for several traits, including Graves' disease and rheumatoid arthritis. To probe the nature of this inflation, we draw Manhattan plots for S-PrediXcan (eQTL data: GTEx (v8) whole blood), CoMM-S^4 (eQTL data: GTEx (v8) whole blood) and CoMM-S^4 (eQTL data: eQTLGen whole blood), using all available genes in the respective analyses. We also add the GWAS Manhattan plot, using all available SNPs in the BBJ GWAS summary statistics. The GWAS and TWAS Manhattan plots suggest that QQ plot inflation for CoMM-S^4 is driven by inflation in the GWAS summary statistics: small p-values in the CoMM-S^4 analyses occur in the same regions as the small p-values in the GWAS dataset (Figures S17-S25).

	No. of associated genes	
Trait	CoMM-S^4	S-PrediXcan
Graves' disease	151	33
Rheumatoid arthritis	159	31
Cerebral aneurysm	1	2
Congestive heart failure	1	0
Ischemic stroke	21	2
Peripheral artery disease	11	0
Osteoporosis	2	0
Chronic hepatitis B	94	7
Chronic hepatitis C	55	3

Table S2. The number of statistically significant genes ($p < 5 \times 10^{-6}$) for the 9 Biobank Japan traits identified by CoMM-S^4 and S-PrediXcan. CoMM-S^4 uses eQTL summary statistics for GTEx (v8) whole blood obtained from the eQTL Catalog; S-PrediXcan uses elastic-net prediction weights for GTEx (v8) whole blood obtained from PredictDB. Only genes common to both analyses were considered for the above table.

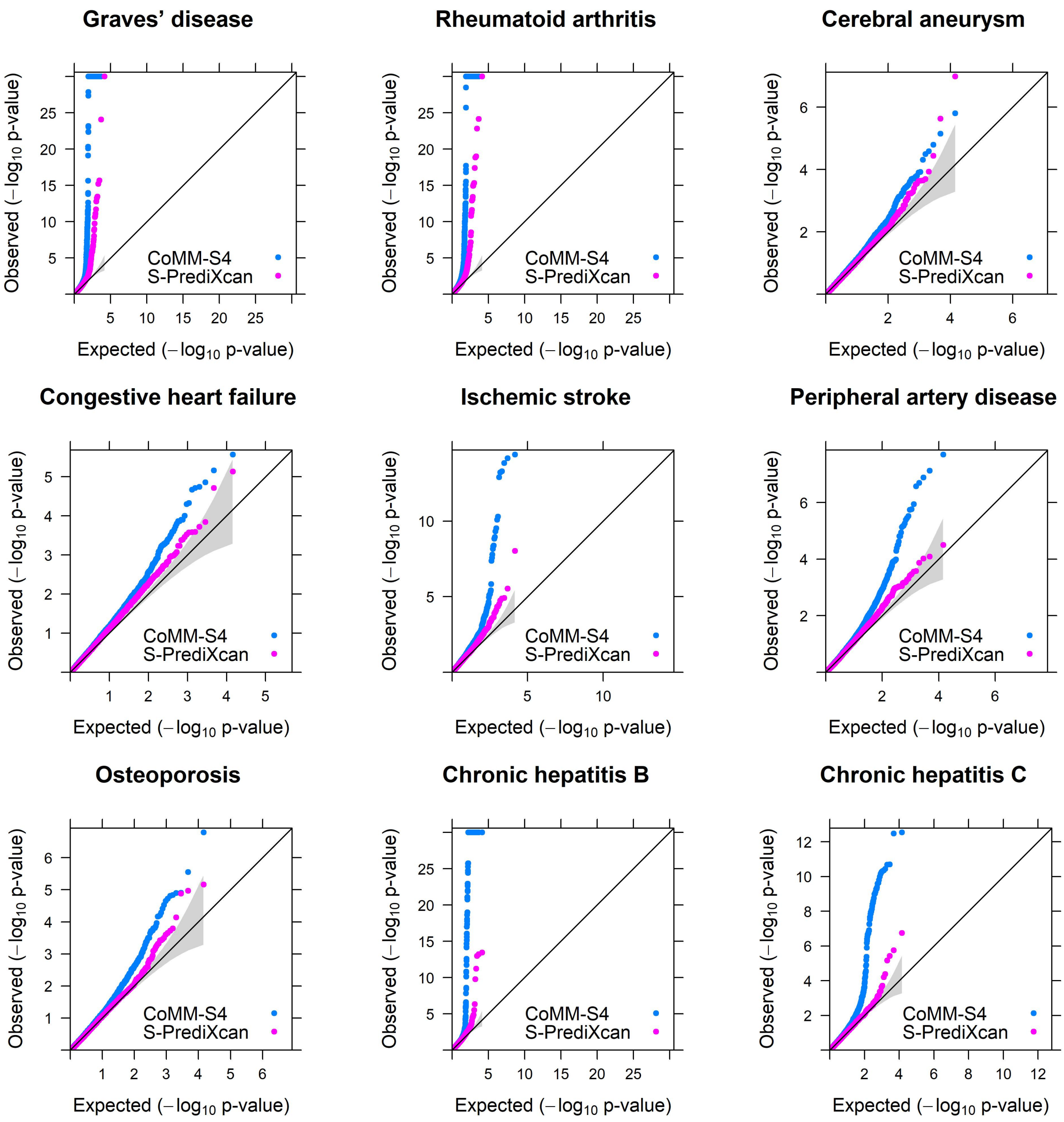

Figure S16: QQ plot comparison for CoMM-S4 and S-Predixcan, in the analyses of 9 Biobank Japan traits. CoMM-S^4 uses eQTL summary statistics for GTEx (v8) whole blood obtained from the eQTL Catalog; S-PrediXcan uses elastic-net prediction weights for GTEx (v8) whole blood obtained from PredictDB. The QQ plots include only genes that are common to both analyses.

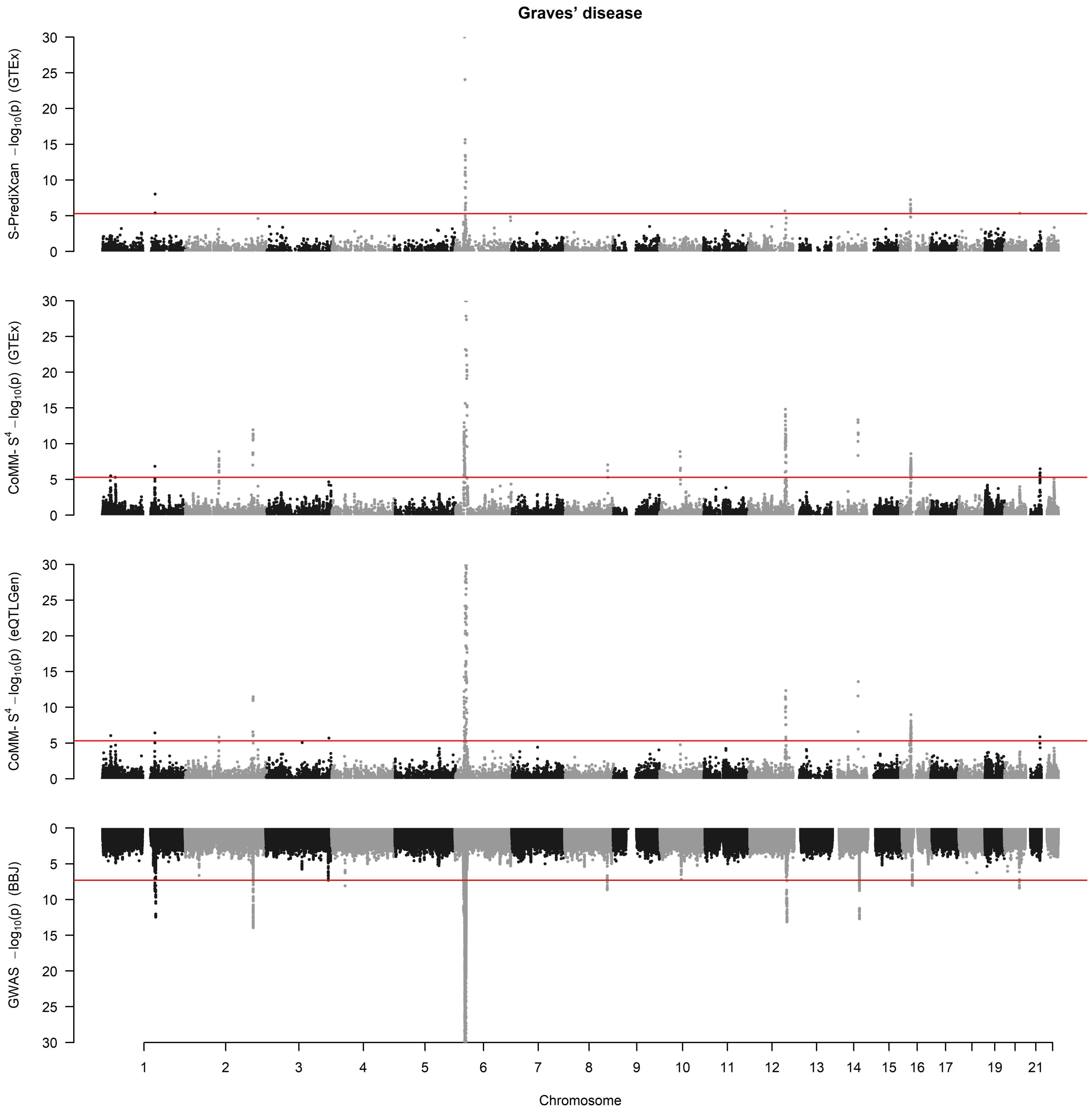

Figure S17: GWAS and TWAS Manhattan plots for Graves' disease. From top to bottom: S-PrediXcan results (eQTL: GTEx (v8) whole blood, GWAS: BBJ), CoMM-S^4 results (eQTL: GTEx (v8) whole blood, GWAS: BBJ), CoMM-S^4 results (eQTL: eQTLGen whole blood, GWAS: BBJ), BBJ GWAS results. The p-value thresholds for association in GWAS and TWAS are 5×10^{-8} and 5×10^{-6}, respectively.

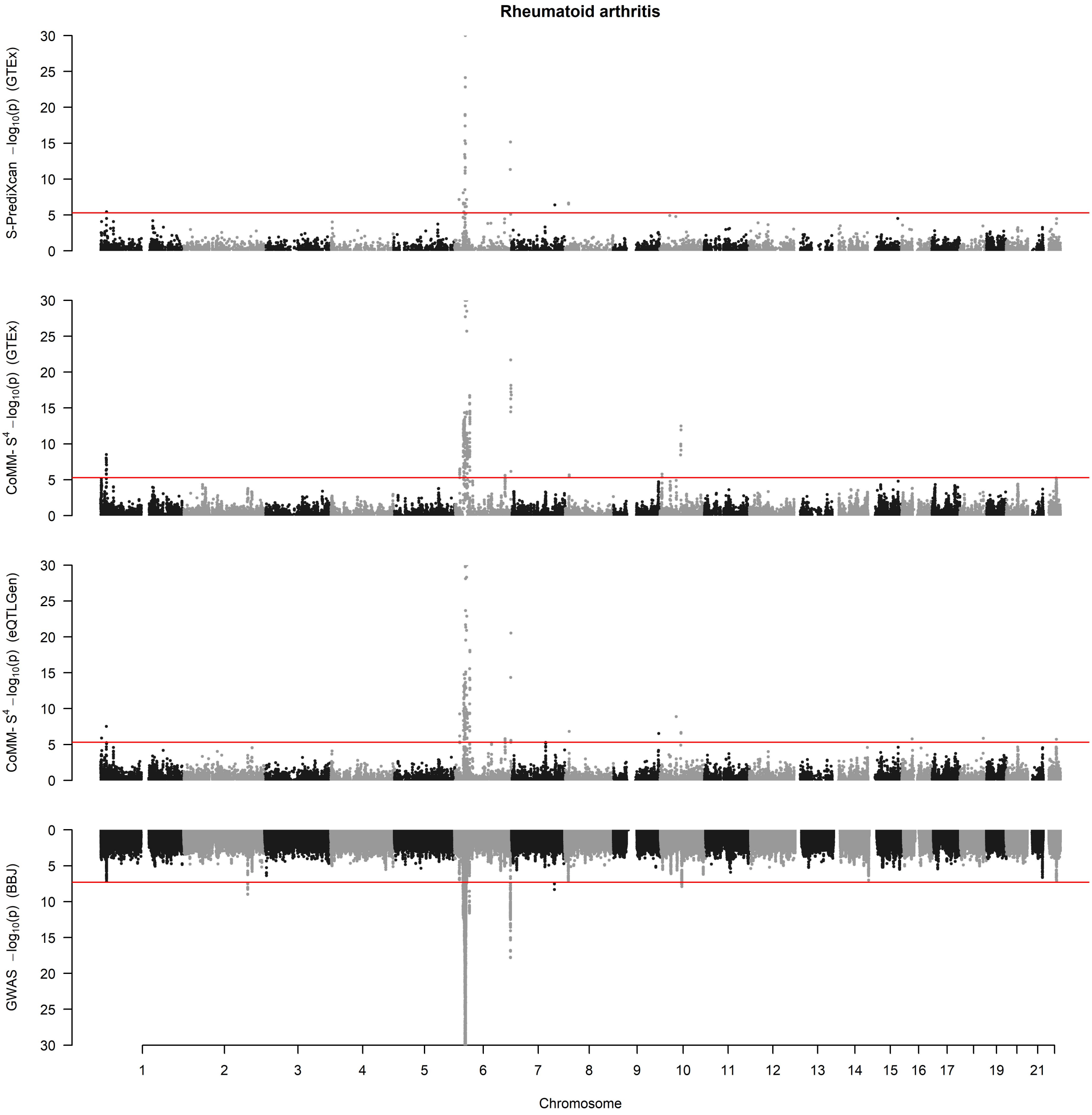

Figure S18: GWAS and TWAS Manhattan plots for rheumatoid arthritis. From top to bottom: S-PrediXcan results (eQTL: GTEx (v8) whole blood, GWAS: BBJ), CoMM-S^4 results (eQTL: GTEx (v8) whole blood, GWAS: BBJ), CoMM-S^4 results (eQTL: eQTLGen whole blood, GWAS: BBJ), BBJ GWAS results. The p-value thresholds for association in GWAS and TWAS are 5×10^{-8} and 5×10^{-6}, respectively.

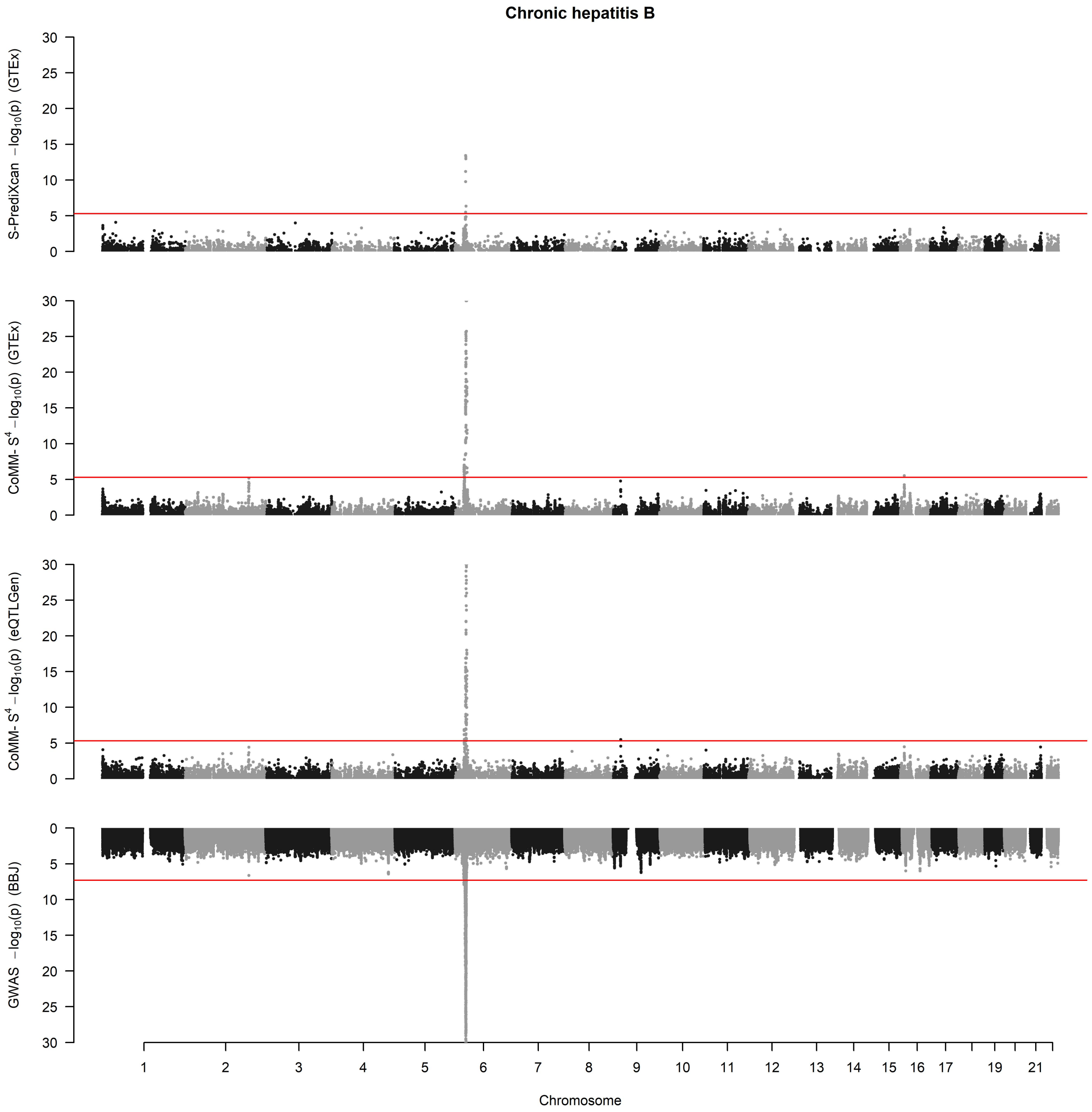

Figure S19: GWAS and TWAS Manhattan plots for chronic hepatitis B. From top to bottom: S-PrediXcan results (eQTL: GTEx (v8) whole blood, GWAS: BBJ), CoMM-S^4 results (eQTL: GTEx (v8) whole blood, GWAS: BBJ), CoMM-S^4 results (eQTL: eQTLGen whole blood, GWAS: BBJ), BBJ GWAS results. The p-value thresholds for association in GWAS and TWAS are 5×10^{-8} and 5×10^{-6}, respectively.

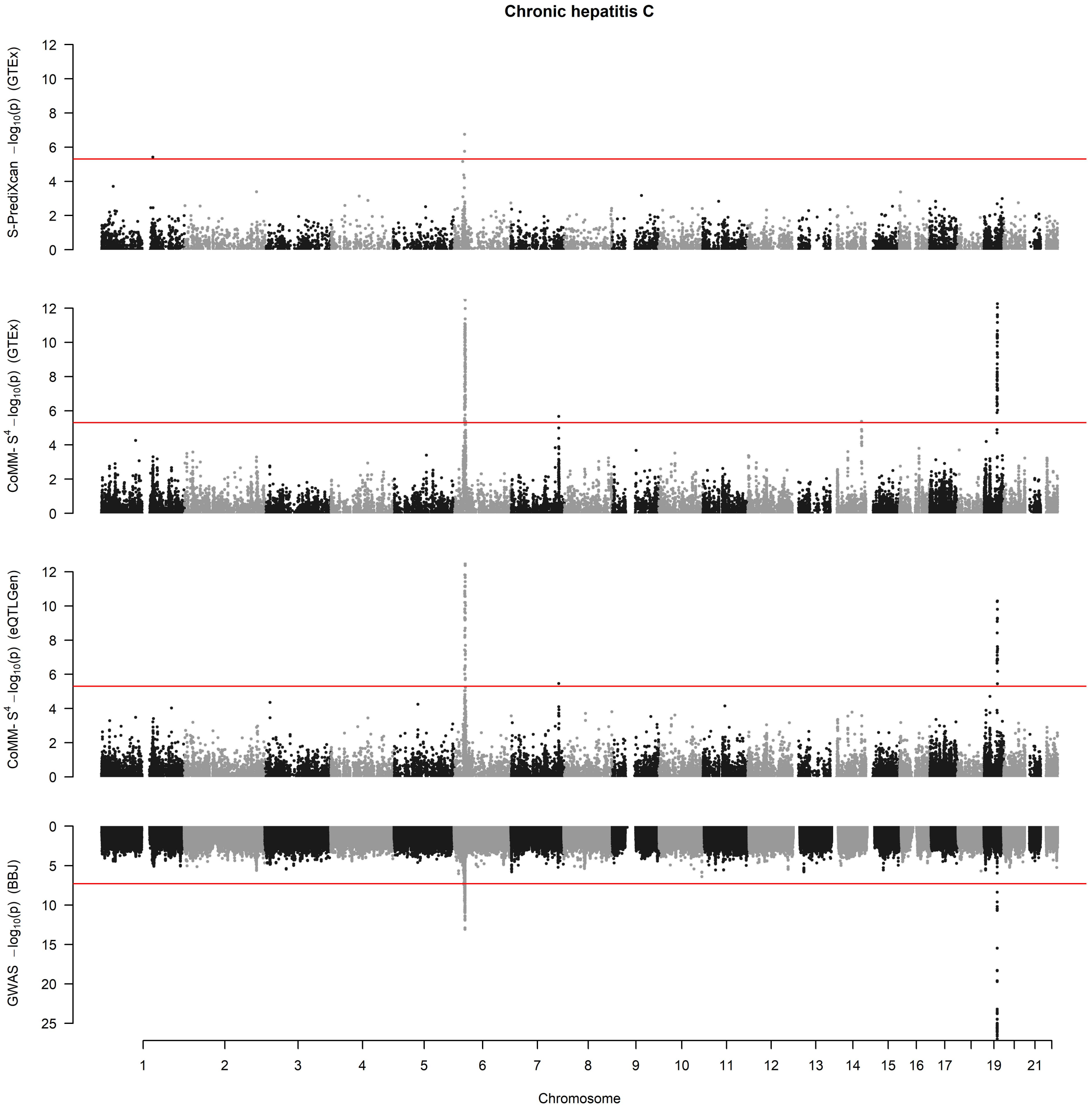

Figure S20: GWAS and TWAS Manhattan plots for chronic hepatitis C. From top to bottom: S-PrediXcan results (eQTL: GTEx (v8) whole blood, GWAS: BBJ), CoMM-S^4 results (eQTL: GTEx (v8) whole blood, GWAS: BBJ), CoMM-S^4 results (eQTL: eQTLGen whole blood, GWAS: BBJ), BBJ GWAS results. The p-value thresholds for association in GWAS and TWAS are 5×10^{-8} and 5×10^{-6}, respectively.

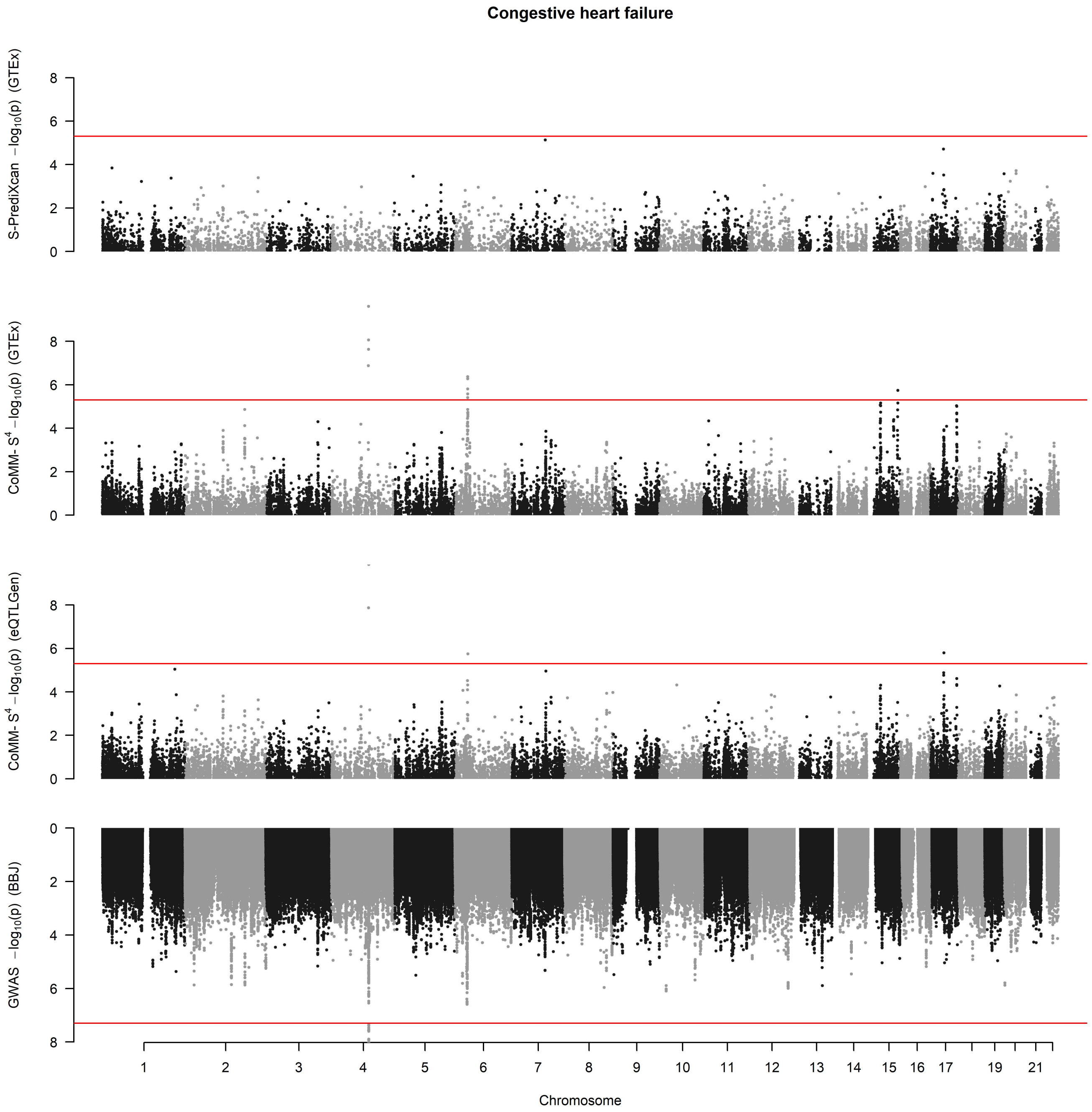

Figure S21: GWAS and TWAS Manhattan plots for congestive heart failure. From top to bottom: S-PrediXcan results (eQTL: GTEx (v8) whole blood, GWAS: BBJ), CoMM-S^4 results (eQTL: GTEx (v8) whole blood, GWAS: BBJ), CoMM-S^4 results (eQTL: eQTLGen whole blood, GWAS: BBJ), BBJ GWAS results. The p-value thresholds for association in GWAS and TWAS are 5×10^{-8} and 5×10^{-6}, respectively.

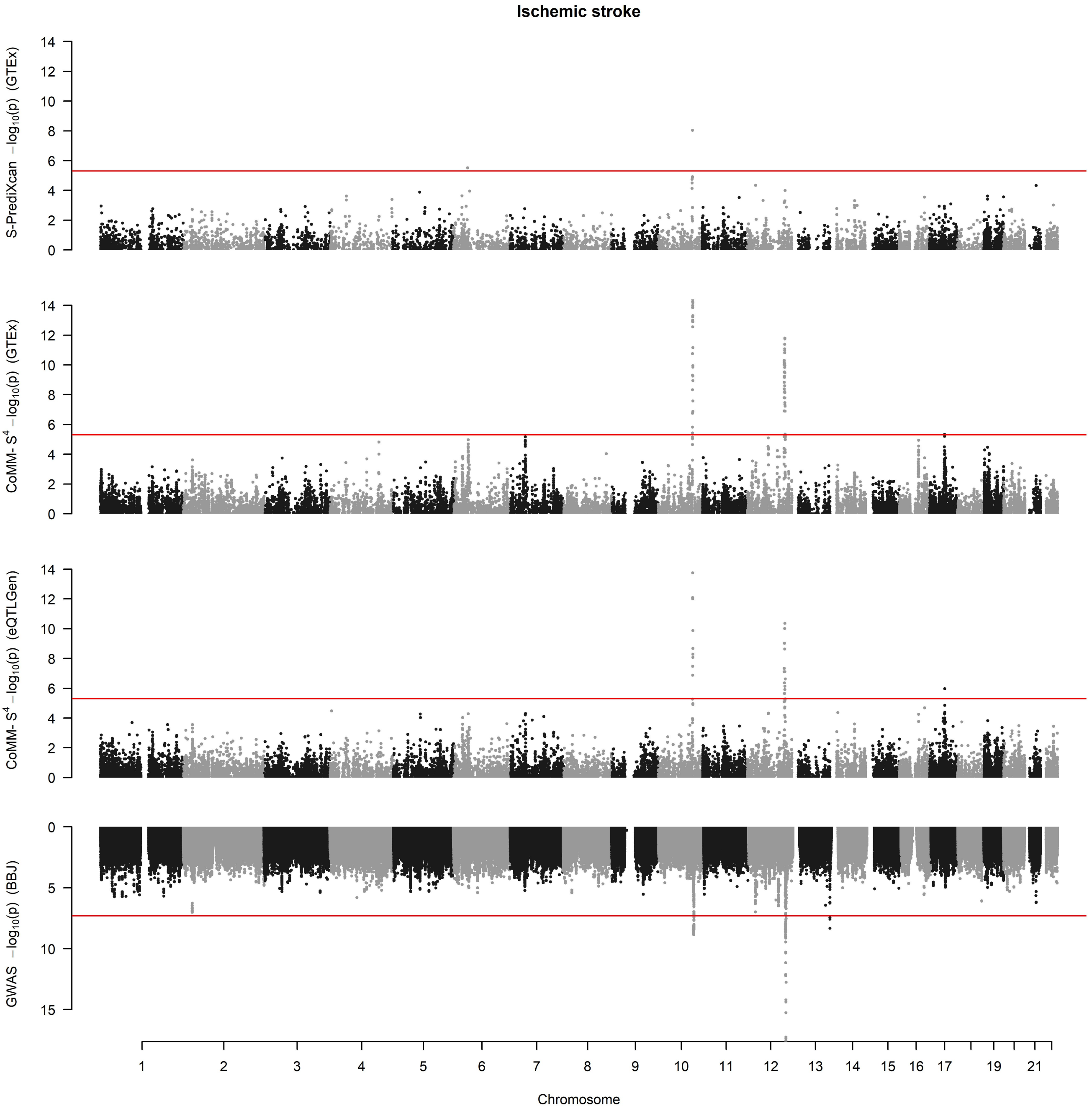

Figure S22: GWAS and TWAS Manhattan plots for ischemic stroke. From top to bottom: S-PrediXcan results (eQTL: GTEx (v8) whole blood, GWAS: BBJ), CoMM-S^4 results (eQTL: GTEx (v8) whole blood, GWAS: BBJ), CoMM-S^4 results (eQTL: eQTLGen whole blood, GWAS: BBJ), BBJ GWAS results. The p-value thresholds for association in GWAS and TWAS are 5×10^{-8} and 5×10^{-6}, respectively.

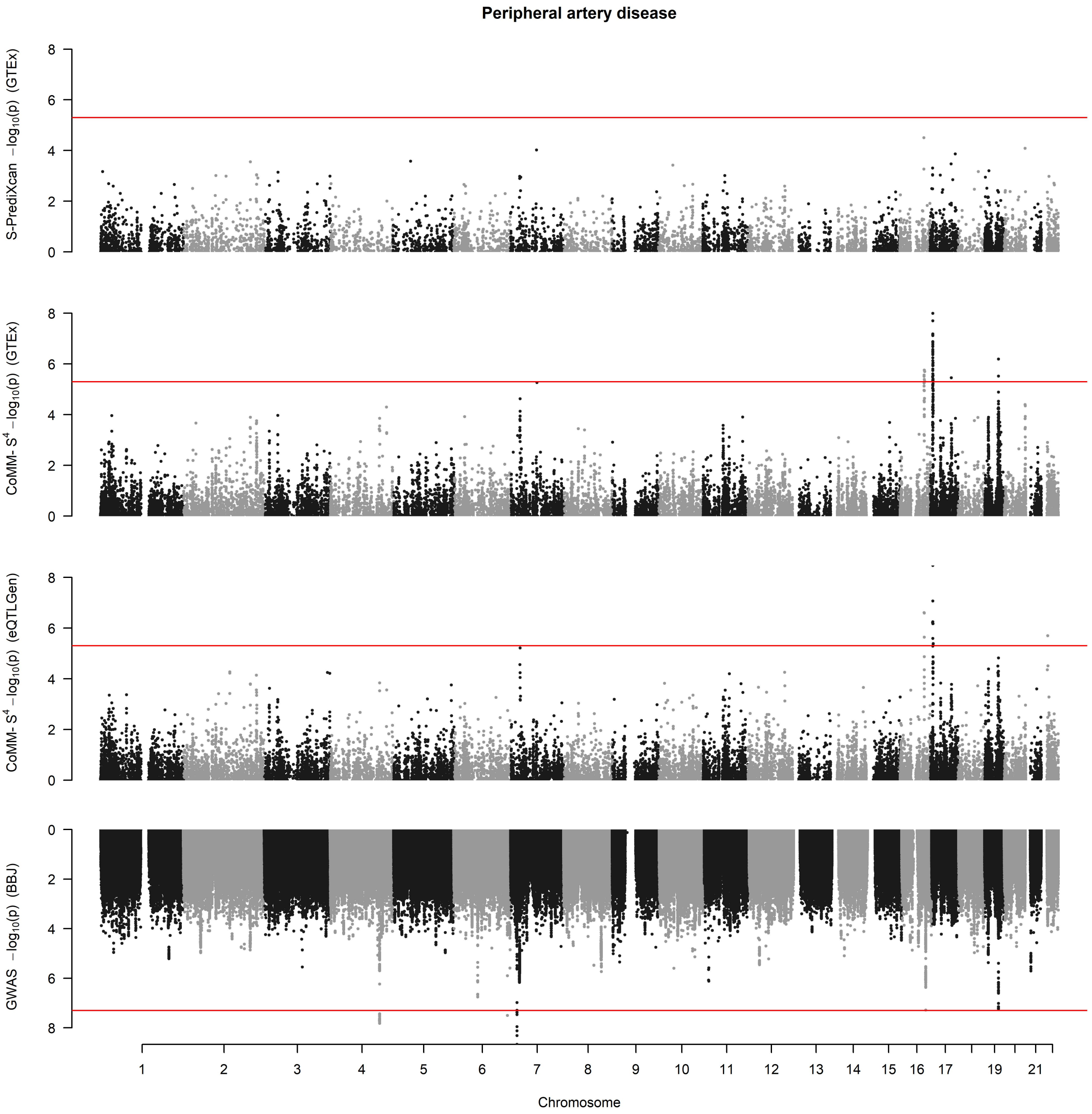

Figure S23: GWAS and TWAS Manhattan plots for peripheral artery disease. From top to bottom: S-PrediXcan results (eQTL: GTEx (v8) whole blood, GWAS: BBJ), CoMM-S^4 results (eQTL: GTEx (v8) whole blood, GWAS: BBJ), CoMM-S^4 results (eQTL: eQTLGen whole blood, GWAS: BBJ), BBJ GWAS results. The p-value thresholds for association in GWAS and TWAS are 5×10^{-8} and 5×10^{-6}, respectively.

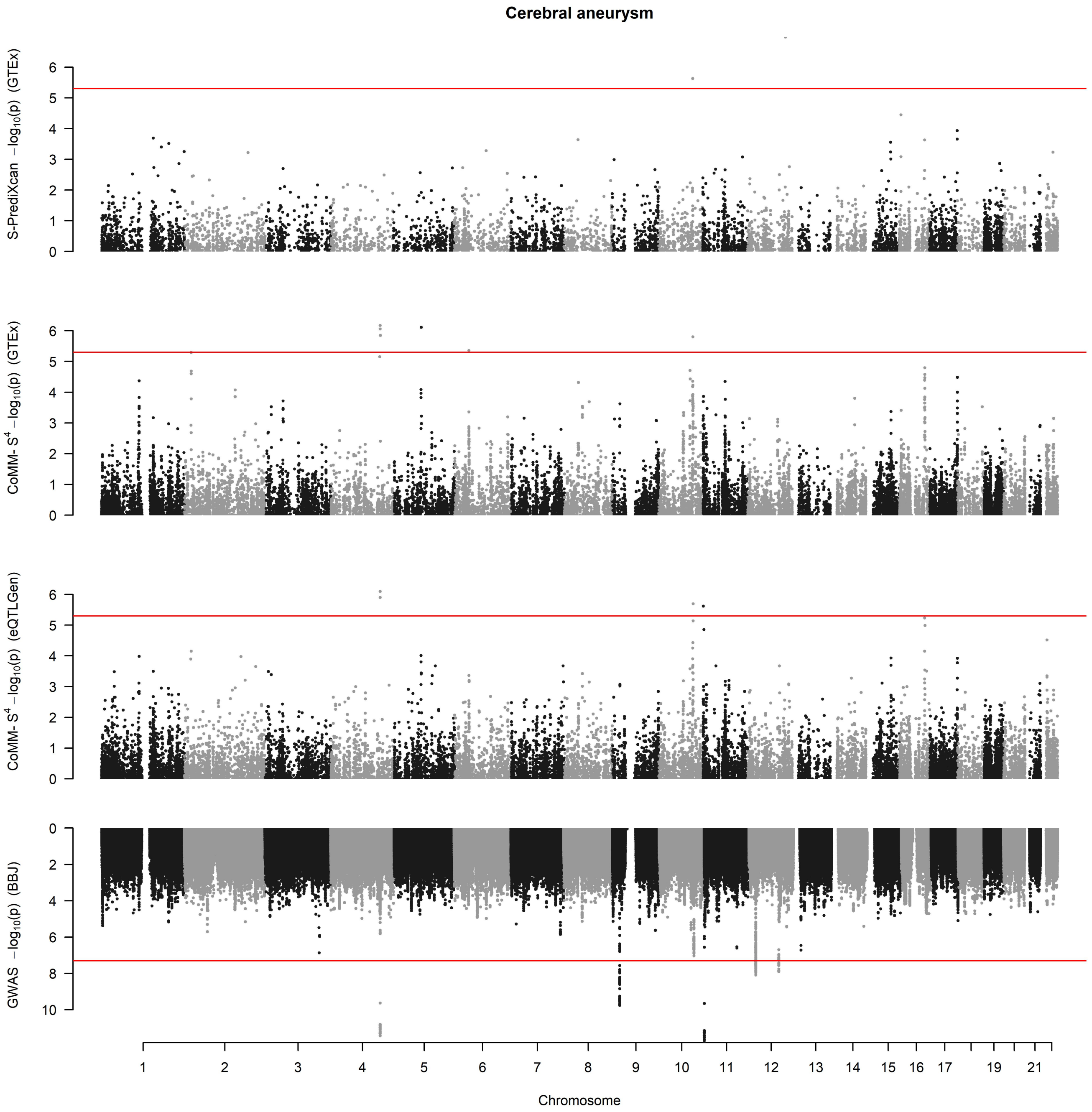

Figure S24: GWAS and TWAS Manhattan plots for cerebral aneurysm. From top to bottom: S-PrediXcan results (eQTL: GTEx (v8) whole blood, GWAS: BBJ), CoMM-S^4 results (eQTL: GTEx (v8) whole blood, GWAS: BBJ), CoMM-S^4 results (eQTL: eQTLGen whole blood, GWAS: BBJ), BBJ GWAS results. The p-value thresholds for association in GWAS and TWAS are 5×10^{-8} and 5×10^{-6}, respectively.

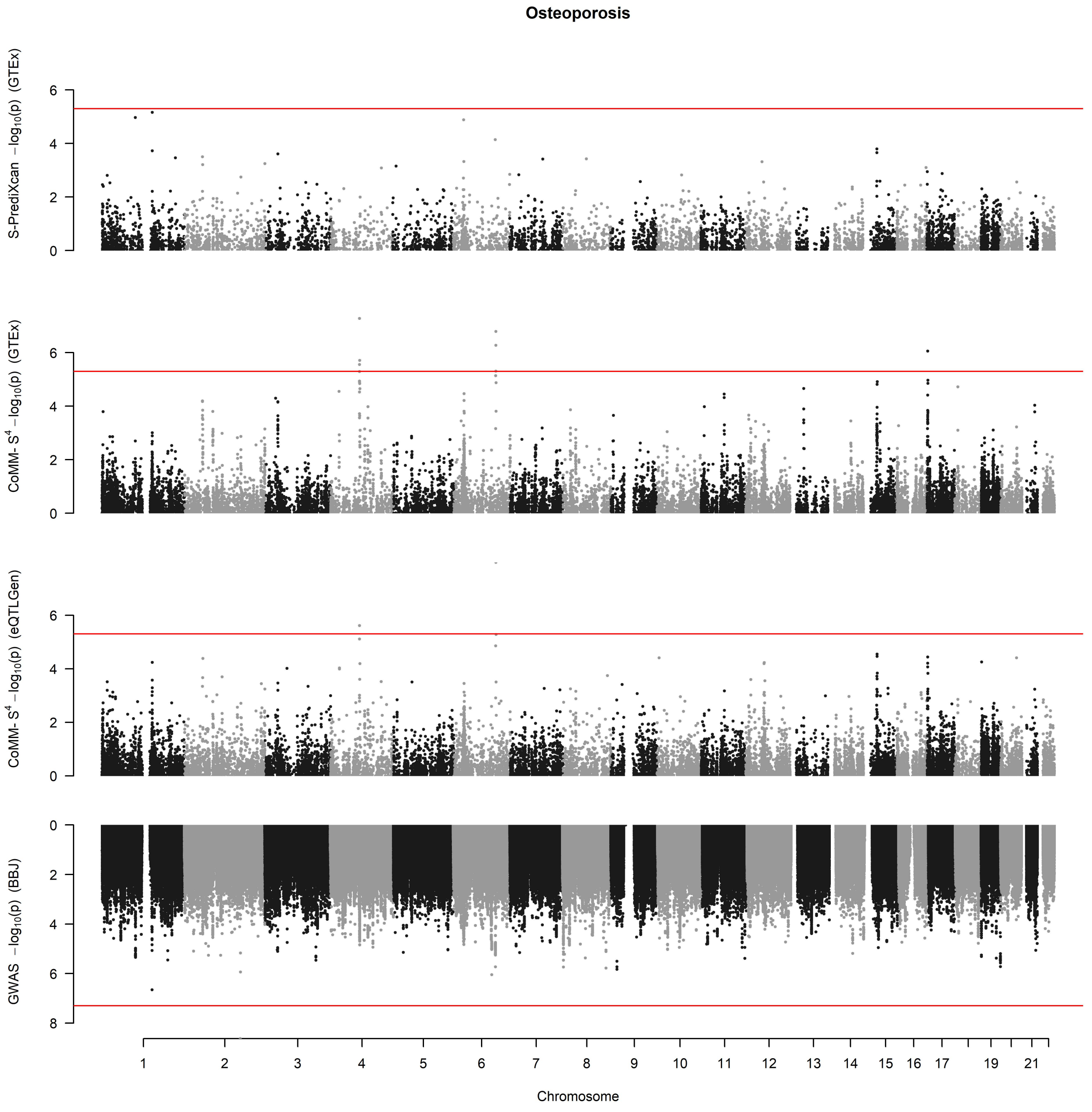

Figure S25: GWAS and TWAS Manhattan plots for osteoporosis. From top to bottom: S-PrediXcan results (eQTL: GTEx (v8) whole blood, GWAS: BBJ), CoMM-S^4 results (eQTL: GTEx (v8) whole blood, GWAS: BBJ), CoMM-S^4 results (eQTL: eQTLGen whole blood, GWAS: BBJ), BBJ GWAS results. The p-value thresholds for association in GWAS and TWAS are 5×10^{-8} and 5×10^{-6}, respectively.

Biological process	p-value	FDR
interferon-gamma-mediated signaling pathway (GO:0060333)	6.86×10^{-10}	8.91×10^{-7}
cellular response to interferon-gamma (GO:0071346)	1.55×10^{-8}	1.01×10^{-5}
antigen processing and presentation of exogenous peptide antigen via MHC class I, TAP-independent (GO:0002480)	2.89×10^{-8}	1.02×10^{-5}
antigen processing and presentation of peptide antigen via MHC class I (GO:0002474)	3.14×10^{-8}	1.02×10^{-5}
antigen processing and presentation of exogenous peptide antigen via MHC class II (GO:0019886)	2.54×10^{-6}	5.13×10^{-4}
antigen processing and presentation of exogenous peptide antigen (GO:0002478)	2.54×10^{-6}	5.13×10^{-4}
antigen processing and presentation of peptide antigen via MHC class II (GO:0002495)	2.76×10^{-6}	5.13×10^{-4}
antigen processing and presentation of exogenous peptide antigen via MHC class I, TAP-dependent (GO:0002479)	3.23×10^{-6}	5.24×10^{-4}
antigen processing and presentation of exogenous peptide antigen via MHC class I (GO:0042590)	4.35×10^{-6}	6.28×10^{-4}
immune response-regulating cell surface receptor signaling pathway (GO:0002768)	6.25×10^{-6}	8.11×10^{-4}
vesicle fusion with endoplasmic reticulum-Golgi intermediate compartment (ERGIC) membrane (GO:1990668)	3.32×10^{-5}	3.92×10^{-3}
positive regulation of apoptotic cell clearance (GO:2000427)	5.76×10^{-5}	6.24×10^{-3}
regulation of apoptotic cell clearance (GO:2000425)	9.14×10^{-5}	9.13×10^{-3}
positive regulation of phagocytosis (GO:0050766)	9.85×10^{-5}	9.14×10^{-3}
cellular response to unfolded protein (GO:0034620)	1.25×10^{-4}	9.80×10^{-3}
cellular response to type I interferon (GO:0071357)	1.28×10^{-4}	9.80×10^{-3}
type I interferon signaling pathway (GO:0060337)	1.28×10^{-4}	9.80×10^{-3}
cytokine-mediated signaling pathway (GO:0019221)	2.43×10^{-4}	1.75×10^{-2}
cellular response to topologically incorrect protein (GO:0035967)	2.86×10^{-4}	1.95×10^{-2}
positive regulation of cytokine production (GO:0001819)	3.40×10^{-4}	2.21×10^{-2}
regulation of protein modification by small protein conjugation or removal (GO:1903320)	5.63×10^{-4}	3.48×10^{-2}
T cell receptor signaling pathway (GO:0050852)	8.06×10^{-4}	4.64×10^{-2}
positive regulation of T cell proliferation (GO:0042102)	8.22×10^{-4}	4.64×10^{-2}

Table S3. Over-representation analysis for Graves' disease, showing only GO biological processes with FDR < 0.05.

Biological process	p-value	FDR
interferon-gamma-mediated signaling pathway (GO:0060333)	$1.20{\times}10^{-11}$	$1.49{\times}10^{-8}$
antigen processing and presentation of exogenous peptide antigen via MHC class II (GO:0019886)	$4.21{\times}10^{-11}$	$1.49{\times}10^{-8}$
antigen processing and presentation of exogenous peptide antigen (GO:0002478)	$4.21{\times}10^{-11}$	$1.49{\times}10^{-8}$
antigen processing and presentation of peptide antigen via MHC class II (GO:0002495)	$4.81{\times}10^{-11}$	$1.49{\times}10^{-8}$
cellular response to interferon-gamma (GO:0071346)	$4.15{\times}10^{-10}$	$1.03{\times}10^{-7}$
T cell receptor signaling pathway (GO:0050852)	$2.71{\times}10^{-8}$	$5.61{\times}10^{-6}$
antigen receptor-mediated signaling pathway (GO:0050851)	$4.96{\times}10^{-6}$	$8.79{\times}10^{-4}$
regulation of protein modification by small protein conjugation or removal (GO:1903320)	$1.24{\times}10^{-5}$	$1.92{\times}10^{-3}$
positive regulation of apoptotic cell clearance (GO:2000427)	$4.27{\times}10^{-5}$	$5.88{\times}10^{-3}$
regulation of apoptotic cell clearance (GO:2000425)	$6.78{\times}10^{-5}$	$8.40{\times}10^{-3}$
cellular response to unfolded protein (GO:0034620)	$8.46{\times}10^{-5}$	$9.53{\times}10^{-3}$
response to unfolded protein (GO:0006986)	$9.88{\times}10^{-5}$	$1.02{\times}10^{-2}$
positive regulation of cytokine production (GO:0001819)	$1.51{\times}10^{-4}$	$1.44{\times}10^{-2}$
regulation of cytokine production (GO:0001817)	$1.77{\times}10^{-4}$	$1.51{\times}10^{-2}$
cellular response to topologically incorrect protein (GO:0035967)	$1.94{\times}10^{-4}$	$1.51{\times}10^{-2}$
immune response-regulating cell surface receptor signaling pathway (GO:0002768)	$1.95{\times}10^{-4}$	$1.51{\times}10^{-2}$
nucleosome organization (GO:0034728)	$2.19{\times}10^{-4}$	$1.55{\times}10^{-2}$
antigen processing and presentation of peptide antigen via MHC class I (GO:0002474)	$2.25{\times}10^{-4}$	$1.55{\times}10^{-2}$
protein-DNA complex assembly (GO:0065004)	$4.71{\times}10^{-4}$	$2.99{\times}10^{-2}$
nucleosome assembly (GO:0006334)	$4.83{\times}10^{-4}$	$2.99{\times}10^{-2}$
positive regulation of T cell proliferation (GO:0042102)	$5.21{\times}10^{-4}$	$3.08{\times}10^{-2}$
cytokine-mediated signaling pathway (GO:0019221)	$5.70{\times}10^{-4}$	$3.21{\times}10^{-2}$
chromatin assembly (GO:0031497)	$6.99{\times}10^{-4}$	$3.77{\times}10^{-2}$
chaperone-mediated protein complex assembly (GO:0051131)	$7.66{\times}10^{-4}$	$3.96{\times}10^{-2}$
positive regulation of phagocytosis (GO:0050766)	$8.23{\times}10^{-4}$	$4.08{\times}10^{-2}$

Table S4. Over-representation analysis for rheumatoid arthritis, showing only GO biological processes with FDR < 0.05.

Biological process	p-value	FDR
antigen processing and presentation of exogenous peptide antigen via MHC class II (GO:0019886)	2.28×10^{-7}	7.89×10^{-5}
antigen processing and presentation of exogenous peptide antigen (GO:0002478)	2.28×10^{-7}	7.89×10^{-5}
antigen processing and presentation of peptide antigen via MHC class II (GO:0002495)	2.47×10^{-7}	7.89×10^{-5}
positive regulation of apoptotic cell clearance (GO:2000427)	9.21×10^{-6}	1.83×10^{-3}
cellular response to unfolded protein (GO:0034620)	1.14×10^{-5}	1.83×10^{-3}
T cell receptor signaling pathway (GO:0050852)	1.15×10^{-5}	1.83×10^{-3}
regulation of apoptotic cell clearance (GO:2000425)	1.47×10^{-5}	2.01×10^{-3}
cellular response to topologically incorrect protein (GO:0035967)	2.66×10^{-5}	3.19×10^{-3}
interferon-gamma-mediated signaling pathway (GO:0060333)	9.27×10^{-5}	8.46×10^{-3}
regulation of protein modification by small protein conjugation or removal (GO:1903320)	9.27×10^{-5}	8.46×10^{-3}
positive regulation of cytokine production (GO:0001819)	9.71×10^{-5}	8.46×10^{-3}
positive regulation of phagocytosis (GO:0050766)	1.18×10^{-4}	9.38×10^{-3}
response to unfolded protein (GO:0006986)	1.73×10^{-4}	1.27×10^{-2}
cellular response to heat (GO:0034605)	2.45×10^{-4}	1.68×10^{-2}
antigen receptor-mediated signaling pathway (GO:0050851)	2.81×10^{-4}	1.79×10^{-2}
positive regulation of leukocyte cell-cell adhesion (GO:1903039)	3.28×10^{-4}	1.96×10^{-2}
negative regulation of signal transduction in absence of ligand (GO:1901099)	4.32×10^{-4}	2.30×10^{-2}
negative regulation of extrinsic apoptotic signaling pathway in absence of ligand (GO:2001240)	4.32×10^{-4}	2.30×10^{-2}
chaperone mediated protein folding requiring cofactor (GO:0051085)	4.91×10^{-4}	2.48×10^{-2}
vesicle fusion with endoplasmic reticulum-Golgi intermediate compartment (ERGIC) membrane (GO:1990668)	6.18×10^{-4}	2.69×10^{-2}
regulation of microtubule nucleation (GO:0010968)	6.18×10^{-4}	2.69×10^{-2}
response to endoplasmic reticulum stress (GO:0034976)	6.44×10^{-4}	2.69×10^{-2}
positive regulation of T cell proliferation (GO:0042102)	6.68×10^{-4}	2.69×10^{-2}
regulation of extrinsic apoptotic signaling pathway in absence of ligand (GO:2001239)	7.00×10^{-4}	2.69×10^{-2}
regulation of cytokine production (GO:0001817)	7.01×10^{-4}	2.69×10^{-2}
regulation of NIK/NF-kappaB signaling (GO:1901222)	7.81×10^{-4}	2.88×10^{-2}
positive regulation of tumor necrosis factor-mediated signaling pathway (GO:1903265)	8.62×10^{-4}	3.06×10^{-2}
‘de novo’ posttranslational protein folding (GO:0051084)	9.58×10^{-4}	3.20×10^{-2}
cellular response to interferon-gamma (GO:0071346)	9.68×10^{-4}	3.20×10^{-2}
positive regulation of IRE1-mediated unfolded protein response (GO:1903896)	1.14×10^{-3}	3.65×10^{-2}
positive regulation of cytokine-mediated signaling pathway (GO:0001961)	1.27×10^{-3}	3.92×10^{-2}
regulation of interleukin-8 production (GO:0032677)	1.39×10^{-3}	4.15×10^{-2}

Table S5. Over-representation analysis for chronic hepatitis B, showing only GO biological processes with FDR < 0.05.

REFERENCES

Bishop, C. M. (2006). *Pattern recognition and machine learning* (Springer)

Ishigaki, K., Akiyama, M., Kanai, M., Takahashi, A., Kawakami, E., Sugishita, H., et al. (2020). Large-scale genome-wide association study in a japanese population identifies novel susceptibility loci across different diseases. *Nature genetics* 52, 669–679

Opper, M. and Saad, D. (2001). *Advanced mean field methods: Theory and practice* (MIT press)

Schäfer, J. and Strimmer, K. (2005). A shrinkage approach to large-scale covariance matrix estimation and implications for functional genomics. *Statistical applications in genetics and molecular biology* 4

The 1000 Genomes Project Consortium (2015). A global reference for human genetic variation. *Nature* 526, 68–74

The GTEx Consortium (2020). The gtex consortium atlas of genetic regulatory effects across human tissues. *Science* 369, 1318–1330

The International HapMap 3 Consortium (2010). Integrating common and rare genetic variation in diverse human populations. *Nature* 467, 52–58

Van der Vaart, A. W. (2000). *Asymptotic statistics*, vol. 3 (Cambridge university press)

Võsa, U., Claringbould, A., Westra, H.-J., Bonder, M. J., Deelen, P., Zeng, B., et al. (2018). Unraveling the polygenic architecture of complex traits using blood eqtl meta-analysis. *bioRxiv* , 447367

Zhu, X. and Stephens, M. (2017). Bayesian large-scale multiple regression with summary statistics from genome-wide association studies. *The annals of applied statistics* 11, 1561
